# The skeleton of the green *Iguana iguana* (Squamata: Iguanidae) and its intraspecific morphological variation

**DOI:** 10.1002/ar.70108

**Published:** 2025-12-17

**Authors:** Vieno Rosa, Marcos Vinícius Coelho, Camila Cupello

**Affiliations:** ^1^ Programa de Pós‐Graduação de Ecologia e Evolução Universidade do Estado do Rio de Janeiro Rio de Janeiro Brazil; ^2^ Departamento de Zoologia Universidade do Estado do Rio de Janeiro Rio de Janeiro Brazil; ^3^ Departamento de Arqueologia Universidade do Estado do Rio de Janeiro Rio de Janeiro Brazil

**Keywords:** anatomy, skull, vertebral column, within‐species diversity

## Abstract

The green iguana (*Iguana iguana*) is an iguanine lizard with herbivorous and arboreal habits, whose distribution spans through South America, Central America to the south of North America. Although the genus *Iguana* is well‐known, the species still lacks a comprehensive and up‐to‐date anatomical study, particularly addressing the axial skeleton, including intraspecific variation. This study provides an anatomical revision of the species, providing a detailed redescription of its cranial skeleton and the first comprehensive analysis of its axial skeleton. Here we use X‐ray computed tomography, 3D segmentation and osteological analyses to examine nine specimens. Significant individual variability was observed in the shape and proportions of skull bones, the presence and absence of a neural spine in the atlas vertebra, rib count and caudal vertebrae morphotypes. Notably, the nasal and frontal bones exhibited shape variation related with specimen size. The caudal vertebrae exhibited considerable variation across specimens, presenting seven distinct morphotypes with consistent sequence but individual variation in their quantity and presence. Moreover, the caudal vertebrae variability contrasts with the previously known caudal pattern for the species. These findings expand our understanding of the morphological diversity within *I. iguana*.

## 1 INTRODUCTION

Iguania is a highly diverse clade within Squamata, comprising over 1900 extant species and divided into the Acrodonta and Iguanidae (=Pleurodonta sensu Schulte et al., 2003) (Paparella & Caldwell, 2021; Smith, 2009). The earliest fossil records of non‐acrodontan iguanians are from the Late Cretaceous of Central Asia and North America (DeMar et al., 2017; Keqin & Norell, 2000; Scarpetta, 2024; Smith, 2009). During this Period, it is hypothesized that stem iguanids migrated from East Asia to North America, leading to the current pattern in which Acrodonta are restricted to the Old World, while Iguanidae are predominantly found in the New World, with some exceptions (de Queiroz, 1987; DeMar et al., 2017; Scarpetta et al., 2025; Smith, 2009; Welt & Raxworthy, 2022). Iguanidae is a large and diverse family, comprising more than 600 species within 55 genera, defined by the presence of pleurodont teeth, the anterior inferior alveolar and subnarial arterial foramina separated on the premaxillary process of the maxilla, and the presence of the processus rostralis on the planum anteorbitalis (de Queiroz, 1987; Smith, 2009). Within it lies the suprageneric group recognized as the iguanine genera (de Queiroz, 1987), which includes several well‐known taxa, such as *Ctenosaura*, *Cyclura*, and *Amblyrhynchus*. These genera encompass a wide ecological spectrum, ranging from terrestrial and arboreal to even semimarine habits (de Queiroz, 1987; Oelrich, 1956; Paparella & Caldwell, 2021).

Among the iguanines, the genus *Iguana* Laurenti, 1768 consists of only two described species, *Iguana iguana* Linnaeus, 1758 and *Iguana delicatissima* Laurenti, 1768. The *I. iguana* differs from its sister group by exhibiting a large scale (subtympanic plate) separated by at most 12 small scales from the ventral margin of the tympanum; a frontal bone confined within the orbits; a septomaxilla articulating with the ventral margin of the nasals; and the presence of autotomy in some caudal vertebrae (Conrad & Norell, 2010; Etheridge, 1967; Lazell, 1973). The *I. iguana*, commonly known as the green lizard or green iguana, is a herbivorous squamate with arboreal habits, although it has also been observed on the ground and even in aquatic environments (Campos & Desbiez, 2013; Lazell, 1973; Lima et al., 2019; Swanson, 2004). Its geographical distribution spans across Central and South America, and it is also found in southern Mexico and southern United States of America (Bock et al., 2022; Campos & Desbiez, 2013); whereas the *I. delicatissima* is restricted to the Lesser Antilles (Bochaton et al., 2016; Conrad & Norell, 2010). The species reaches sexual maturity within 2–3 years, and following copulation, egg‐laying occurs 40 to 50 days later, with groups of more than 60 eggs being deposited (Lima et al., 2019).

The vertebrate skeleton exhibits characteristics related to species phylogeny as well as ecology, which includes adaptations related to posture, locomotion, feeding habits, and even respiration (Janis & Keller, 2001). Most studies on the morphology of the *I. iguana* skeleton focus on components of the skull (Conrad & Norell, 2010; Evans, 2008; Lima et al., 2014; Mohamad et al., 2023; Pérez Alberto et al., 2021), with only a few addressing the ribs (Brainerd et al., 2016) or caudal vertebrae (Etheridge, 1967). Descriptions of the vertebral column in Squamata are generally less common than those of the skull and typically concentrate on broad clades (Tschopp, 2016). Nevertheless, the tail plays a crucial role in locomotion and body posture, while also displaying significant morphological diversity (Tomańska et al., 2024). Its importance is further emphasized in arboreal species such as *I. iguana*, since arboreal habitats have been associated with longer tails compared to terrestrial species, as demonstrated in the iguanian clade Chamaeleonidae (Luger et al., 2019).

Although several studies have analyzed variation among Iguanidae taxa (de Queiroz, 1987; Evans, 2008; Smith, 2009), data on intraspecific variation remain limited. This contrasts with the general condition in reptiles, which exhibit a highly variable number of elements per vertebral region, sometimes even displaying slight intraspecific variation (Tschopp, 2016). While investigating osteological differentiation within the genus *Iguana*, Bochaton et al. (2016) noted a high degree of intraspecific variation in *I. iguana*. Similarly, Conrad and Norell (2010) reported that *I. iguana* exhibits greater external morphological variability compared to *I. delicatissima*. Studying such morphological variation is essential for understanding species ecology, anatomy, and the fossil record (Lai & Scarpetta, 2024), even in a species as well‐known as *I. iguana*.

Here, we utilize three‐dimensional models based on X‐ray computed tomography scans, in addition to prepared skeletons, to analyze and redescribe the morphology of the cranial and vertebral column structure of the green iguana, including its vertebral architecture and ribs. This study not only provides a detailed cranial description but also offers the first comprehensive analysis of the axial skeleton of *I. iguana*, emphasizing morphological variations within both the skull and axial skeleton.

## 2 MATERIALS AND METHODS

### 2.1 Specimen information

Nine specimens from different collections were studied, including openly available CT scans, 3D reconstructions and mechanically prepared skeletons, as detailed in Table 1.

**TABLE 1:** Specimens used in this article.

Collection id	Institution	Preservation	Body part	Sex	Ontogenetic stage
DZ UERJ	Universidade do Estado do Rio de Janeiro	Osteological	Complete	–	Juvenile
Ig01	Brown University	CT scan	Complete	Male	Adult
Ig02	Brown University	CT scan	Rib cage	Male	–
Ig03	Brown University	CT scan	Rib cage	Female	Adult
Ig04	Brown University	CT scan	Complete	Male	Adult
Ig05	Brown University	CT scan	Presacral skeleton (skull incomplete)	Male	Adult
MNRJ 12124	Museu Nacional	osteological	Skull	–	Adult
OUVC 10677	Ohio University	3D reconstruction	Skull and neck	–	Late juvenile
UF:Herp:181922	Florida Museum of Natural History, University of Florida	CT scan	Complete	Female	Juvenile

### 2.2 X‐ray tomography

The five computed tomography scans (Ig01, Ig02, Ig03, Ig04 and Ig05) were available through the X‐ray Motion Analysis Research Portal (XMAPortal; https://xmaportal.org) originally used in the study Brainerd et al. (2016). The specimen OUVC 10677 was available in Sketchfab under the license CC by 4.0 (https://creativecommons.org/licenses/by/4.0/). The specimen UF:Herp:181922 was available with no known Copyright in Morphosource (https://www.morphosource.org) by Blackburn et al. (2024). CT scan images were available as 16‐bit TIFF stacks, which were pre‐processed in ImageJ software and converted to 8‐bit TIFF stacks. For all the specimens (Ig01, Ig02, Ig03, Ig04, Ig05 and UF:Herp:181922), segmentation and three‐dimensional rendering were realized at the workstations from the Departamento de Arqueologia and the Departamento de Zoologia of the Universidade do Estado do Rio de Janeiro (UERJ) using the software MIMICS Innovation Suite 25.0 (Materialise). All the scan information is provided at Table S1. Additionally, photogrammetry 3D surface models of two specimens (MNRJ 12124 and DZ UERJ) were generated using Agisoft Metashape 2.1.2 (Agisoft) software. Specimen UF:Herp:181922 was selected for the detailed description of the skull due to the superior quality of its CT scan, although it is important to note that the tomography resolution of all specimens was limited. The specimen Ig04 was chosen for the description of the postcranial skeletal elements because it was the only adult specimen with a complete skeletal set. The left forelimb and hindlimb were segmented for Figures 12 and 13, respectively, on account of their greater completeness compared to the contralateral limbs.

### 2.3 Nomenclature

In the present study, all specimens were identified as *I. iguana* based on diagnostic characters described by Conrad and Norell (2010) and Bochaton et al. (2016). The anatomical description of the skull is based on the nomenclature of Evans (2008). The descriptions of the vertebrae anatomy follows the works of Tschopp (2016), with the exception of Williston (1925) for the hypapophysis nomenclature, Veronese and Krause (1997) for the cotyle and condyle nomenclature, as well as Etheridge (1967) for caudal vertebrae anatomy. The rib description is based on Romer (1950), but also Brainerd et al. (2016) for the rib joints nomenclature. The pectoral and pelvic girdle, as well as the forelimb and hindlimb, is based on the nomenclature of Kardong (2012). The palmar sesamoid nomenclature is based on Fontanarrosa and Abdala (2014).

The caudal vertebrae are classified into morphotypes based on Etheridge (1967), with the exception that the neural spine and haemal arch are also considered in this study. Therefore, we propose seven morphotypes, where the first morphotype of Etheridge (1967) corresponds to morphotype 1 and 2 in the present classification. The division of the caudal segment into proximal, transitional, and distal regions follows Tomańska et al. (2024).

## 3 RESULTS

### 3.1 Skull bones

The skull of *I. iguana* is deep and triangular when viewed dorsally (Figure 1). The premaxilla (Px) lies at the most anterior part of the skull, with a slender, triangular shape, and is relatively small compared to other bones, measuring approximately one‐third the size of the nasal (Figures 1a–h and S1a–h). Its anterior margin articulates with the maxilla, while the anterior half of its lateral margin forms the medial portion of the nares. The posterior half of its lateral margin articulates with the nasal. It exhibits five teeth along its ventral margin.

**FIGURE 1:**
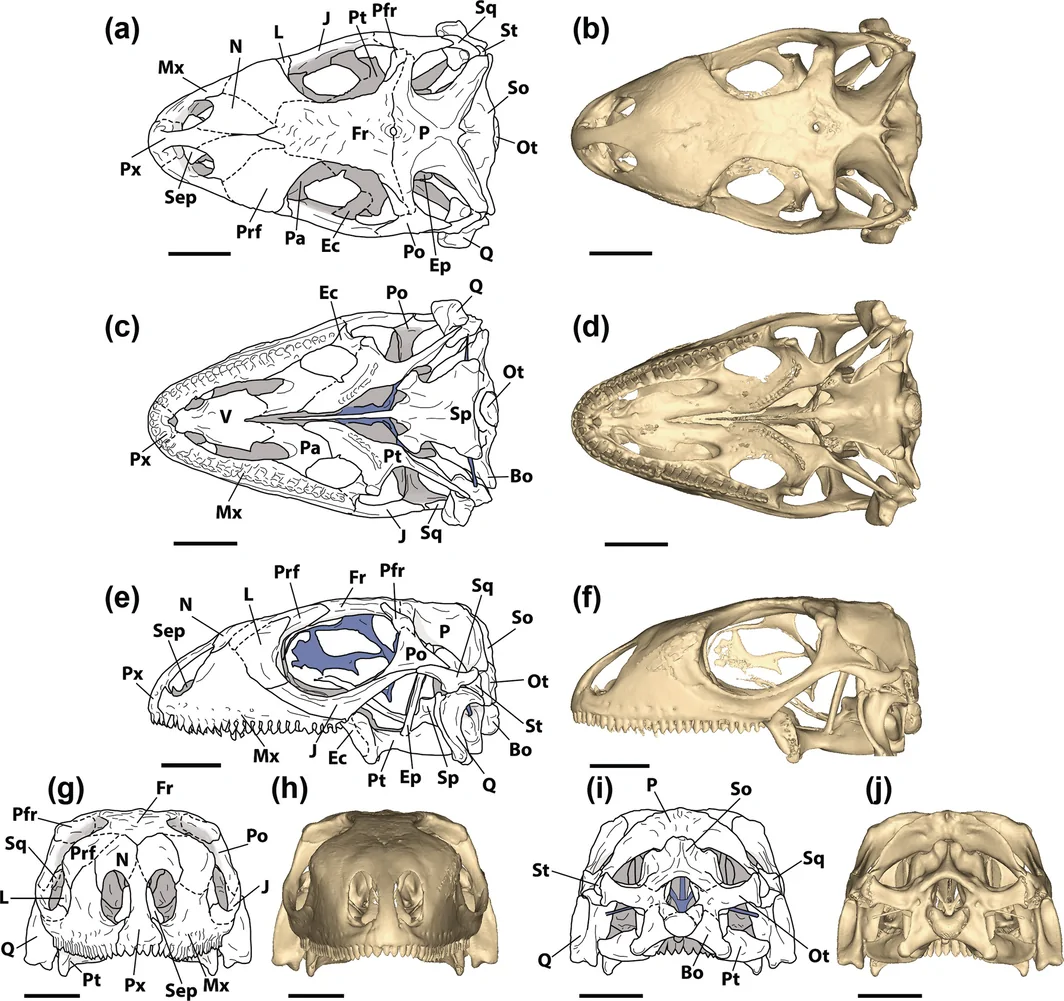
Skull of the UF:Herp:181922 specimen: (a) drawing of dorsal view, (b) dorsal view, (c) drawing of ventral view, (d) ventral view, (e) drawing of left lateral view, (f) left lateral view, (g) drawing of anterior view, (h) anterior view, (i) drawing of posterior view, (j) posterior view. Bo, Basioccipital; Ec, Ectopterygoid; Ep, Epiterygoid; Fr, Frontal; J, Jugal; L, Lacrimal; Mx, Maxilla; N, Nasal; Ot, Otooccipital; P, Parietal; Pa, Palatine; Pfr, Postfrontal; Po, Postorbital; Prf, Prefrontal; Pt, Pterygoid; Px, Premaxilla; Q, Quadrate; Sep, Septomaxilla; So, Supraoccipital; Sp, Sphenoid; Sq, Squamosal; St, Supratemporal; V, Vomer. Dashed lines, inferred sutures based on the literature and other specimens. Blue, cartilaginous structure. Scale bars, 1 cm.

The paired nasals (N) constitute the posterior margin of the nares, articulating anterolaterally with the maxilla, laterally with the prefrontal, posteromedially with the frontal, and anteromedially with the premaxilla (Figures 1a, b, e, h and S1a–f). The nasal exhibits a triangular shape, measuring approximately one‐third the size of the frontal.

The septomaxilla (Sep) is a paired bone located within the nares, positioned posterior to the premaxilla and measuring two‐thirds its size (Figures 1a, b, e–h and S1e,f). It constrains a thin, slightly curved, plate‐like structure, with a postero‐ventral process that extends diagonally posteriorly to articulate with the vomer. Anteriorly, the septomaxilla articulates with the premaxillary process of the maxilla, where each septomaxilla displays a flattened, circular process.

The pair of prefrontals (Prf) exhibit a triangular shape and articulate medially with the nasal and the frontal, anteroventrally with the maxilla, ventrally with the lacrimal, ventrolaterally with the jugal and form the anterolateral margin of the orbit (Figures 1a, b, e–h and S1a–f). The prefrontal exhibits an average relative size when compared to other bones such as the nasal, lacrimal, and postfrontal, measuring two‐thirds of the length of the frontal.

The frontal (Fr) is the largest component of the skull roof, measuring one‐fourth the length of the cranium, and forms the medial margin of the orbits (Figures 1a, b, e–h and S1a–h). It is long and broad, with a quadrangular shape, tapering anteriorly and expanding lateroposteriorly, featuring plate‐like ornamentation. It articulates anteriorly with the nasal and premaxilla, laterally with the prefrontal, posteriorly with the parietal, and at its lateral posterior margins with the postfrontals. The frontoparietal suture houses the parietal foramen, which has thick borders, an oval opening, and is half‐formed by the frontal and half by the parietal.

The pair of postfrontals (Pfr) are the smallest bones of the skull roof, small and irregular in shape, and slightly shorter than the premaxilla (Figures 1a–h and S1a–h). They constitute part of the posterior portion of the orbit. The postfrontal articulates anteromedially with the frontal, medially with the parietal and lateroposteriorly with postorbital.

The parietal (P) is situated at the most posterior portion of the skull roof and is relatively large, comprising approximately one‐fourth of the total cranial length. Its surface exhibits distinct ornamentation (Figures 1a, b, e, f, i, j and S1a–h). Anteriorly, the parietal articulates with the frontal via the frontoparietal suture. It forms the medial borders of the upper temporal fenestrae. The parietal is quadrangular in shape, with lateral extensions on both of its anterior and posterior margins, known as the anterolateral and supratemporal processes, respectively, or postparietals. The anterolateral process articulates anterolaterally with the postfrontal and laterally with the postorbital. The supratemporal process articulates anterolaterally with the squamosal, posterolaterally with the otooccipital, and ventrally with the supratemporal. A crest‐like process on its posterior margin articulates with supraoccipital.

The paired maxillae (Mx) are large, with a length equal to half the length of the skull in lateral view, and are scalene triangle shaped (Figures 1a–h and S1a–h). The maxilla is positioned in the lateral region of the skull, separated anteriorly by the premaxilla. It has three external processes: an anterior process that articulates with the premaxilla, a dorsal process that articulates with the nasal, prefrontal, and lacrimal, and a posterior process that articulates laterally with the jugal. Between the anterior and dorsal processes, the maxilla forms the lateral margins of the nares. Inside the skull, it articulates posteriorly in its anterior region with the vomer and septomaxilla by the premaxillary process. It also articulates laterally with the palatine and posteriorly with the ectopterygoid. Its ventral margin features pleurodont teeth, with the bases attached to the ventrolateral surface of the maxilla. Specimen UF:Herp:181922 shows 22 teeth that extend along its ventral margin. In its medial surface, the maxilla exhibits a foramen for the passage of blood vessels that projects through the anterior one‐fourth of the length of the bone.

The lacrimal (L) is a small, slender paired bone, approximately one‐fifth the size of the prefrontal (Figures 1a, b, e, h and S1c–f). It articulates ventrolaterally with the maxilla, dorsoanteriorly with the prefrontal, posteriorly the jugal, and medially with the palatine. The lacrimal composes an anterior portion of the orbit and the jugal (J) forms the curvature of its ventral margin. The jugal is a thin, long, and curved paired bone, with a length approximately equal to that of the frontal (Figures 1a–h and S1a–d, g, h). The jugal articulates laterally with the lacrimal, laterodorsally with the prefrontal, and ventrally with the maxilla along its entire length. Its posterior portion articulates laterodorsally with the postorbital, posteriorly with the squamosal, and medially with the ectopterygoid.

The paired postorbitals (Po) exhibit a triangular shape with slightly elongated, thin processes (Figures 1a–h and S1a–h). Their size is nearly the same as the prefrontal, and they are located at the posterior margin of each orbit. The postorbital articulates dorsally with the postfrontal and parietal, lateroventrally with the jugal, and posteroventrally with the squamosal. Its posterior margin forms the anterior region of the upper temporal fenestra. The squamosal (Sq) is a small paired bone with a relatively fusiform shape, forming the ventral margin of the upper temporal fenestra (Figures 1a–j and S1a–h). Its anterior process articulates with the postorbital and jugal, while its posterior process articulates with the parietal and supratemporal. The squamosal lies ventral to the dorsal margin of the quadrate, and its length is slightly smaller than that of the postorbital.

The supratemporal (St) is a very small, paired bone, about one‐fourth the size of the squamosal, with a trapezoid shape (Figures 1a, b, e, f, i, j and S1e, f). It articulates anteriorly with the squamosal, dorsally with the parietal, posteriorly with the otooccipital, and ventrally with the quadrate.

The quadrate (Q) is a paired bone located in the posterior region of the skull (Figures 1a–j and S1e, f). It exhibits a concave shape with a broad, curved anterior surface, while the posterior surface features a posterior crest, creating two concave fossae. Dorsally, it bears a large, slightly rounded cephalic condyle that articulates with the squamosal, supratemporal, and otooccipital. Ventrally, it presents a mandibular condyle that articulates with the articular and surangular. Medially, the quadrate articulates with the transverse process of the pterygoid.

The otooccipital (Ot) is a paired bone positioned in the posterior region of the skull, also known as the neurocranium (Figures 1a–f, i, j, S1e, f and S2a–j). Its size is approximately half the height and width of this region. The otooccipital features a relatively triangular shape, with an elongation in each vertex. The lateral process is approximately twice the size of the other processes and articulates with the supratemporal, quadrate and parietal bones; in its anterior surface, it articulates with the prootic inside the skull. The dorsal process articulates with the supratemporal, while the ventral process articulates with the basioccipital. The otooccipital contributes to the lateral region of the occipital condyle and the lateral margin of the foramen magnum.

The supraoccipital (So) possesses the same width as the otooccipital, but half its height. It articulates dorsally with the parietal through a process (Figures 1a, b, e, f, i, j and S2a–f, i, j). At the midpoint of this structure, ventral to the parietal bone, lies a small cone‐like process measuring approximately one‐eighteenth of the total of the supraoccipital (Figure S1a–f, i, j). Lateroventrally, it articulates with the two otooccipitals, so its ventral wall forms the dorsal margin of the foramen magnum. Anteriorly, in the region inside the skull, it articulates with the prootic. The supraoccipital features a wide crest in its medial region, molding two lateral, concave, and shallow fossae. The basioccipital (Bo) is of equal size to the otooccipital and is positioned in the neurocranium, extending to the ventral region of the skull (Figures 1c–f, i, j, S1e, f and S2a–j). It creates the ventral margin of the foramen magnum, as well as forms the occipital condyle, and projects two wing‐shaped processes ventrally. Laterally, it articulates with the otooccipital, and in its posterior region, it articulates with the sphenoid.

The sphenoid (Sp) is a bone formed by the fusion of the parabasisphenoid and the basisphenoid (Evans, 2008), constituting one‐fourth of the length of the skull (Figures 1c–f, S1e, f and S2a, j). It presents a semicircular shape and features a long parasphenoid rostrum, which is a slender structure that extends anteromedially to the posterior limit of the maxilla. The posterior concavity articulates with the basioccipital. It articulates laterodorsally through the alar process with the prootic. Additionally, it exhibits two anterolateral basipterygoid processes that articulate with the pterygoid.

The prootic (Pro) composes part of the otic capsule, in the anterior part of the neurocranium, and constitutes one‐third of the total width of the skull (Figures S1e, f and S2a–j). Its shape is triradiate, with three distinct processes: an anterior, a dorsal and a posterior process. The anterior inferior process articulates ventrally with the sphenoid. Between this process and the dorsal process, also known as the alar process, is a curve called the incisura prootica. The prootic articulates posteriorly with the supraoccipital through its posterior surface, extending to the dorsal tip of the alar process. Anterior to the alar process is a curved edge, the crista alaris. The posterior process is the largest process, extending posteriorly and articulating with the anterior facet of the otooccipital. The lateral surface between the alar and the posterior processes is curved and is called the crista prootica.

The pterygoid (Pt) is one of the largest paired bones in the skull of *I. iguana*, extending across half of its ventral region (Figures 1a–j and S1e, f). It consists of three large processes: an anterior process that articulates with the palatine, a lateral process that articulates with the ectopterygoid, and a long posterior process that articulates with the quadrate. Along with the medial margin of the posterior process, it articulates with the basipterygoid process of the sphenoid. Dorsally, the pterygoid articulates with the epipterygoid. The pterygoid is also situated among three openings: the suborbital fenestra, the subtemporal fenestra, and the interpterygoid vacuity. The suborbital fenestra, located below the orbit, is formed by the maxilla, ectopterygoid, palatine and pterygoid bones. The subtemporal fenestra is formed by the ectopterygoid, pterygoid and quadrate bones. Lastly, the interpterygoid vacuity is a narrow opening extending from the posterior margin of the vomer to the pterygoid. Additionally, a series of small teeth is aligned diagonally across the central ventral region of the pterygoid, extending from the medial to the lateral margin.

The epipterygoid (Ep) is a thin, elongated paired bone that extends vertically within the cranial cavity, constituting approximately three‐fourths of the skull's height (Figures 1a, b, e, f and S1e, f). Its ventral process articulates with the pterygoid, while its dorsal process articulates with the parietal. The ectopterygoids (Ec) are small and elongated, presenting a rectangular shape and width of one‐fourth of the skull's width (Figures 1a–f and S1e, f). The anterior margin of the ectopterygoid articulates with the maxilla, while its posterior margin articulates with the jugal and the lateral process of the pterygoid. The medial margin contributes to the formation of the suborbital fenestra, while the posterior margin is part of the subtemporal fenestra.

The palatine (Pa) is a paired bone that constitutes approximately two‐seventh of the length of the skull (Figures 1a–d and S1e, f). Its shape is similar to that of the pterygoid, as it also possesses three processes, though shorter. The anterior process articulates with the vomer, the lateral process with the maxilla, lacrimal, jugal and prefrontal bones, while the posterior process articulates with the pterygoid. The choana is a narrow opening formed by the maxilla, vomer and palatine, located between the anterior and lateral processes of the palatine. The suborbital fenestra is situated between the lateral and posterior processes, while the interpterygoid vacuity is present along its medial margin.

The vomer (V), comprising approximately one‐seventh of the skull's length, is the most anterior bone in the ventral region (Figures 1c, d and S1e, f). The two slender, slightly triangular vomer bones articulate medially with each other. Posteriorly, they articulate with the palatine via a straight transverse suture and articulate anteriorly with the maxilla and premaxilla. The lateral margin forms part of the choana, and the posterior point of contact between both vomer bones marks the beginning of the interpterygoid vacuity.

The dentary (D) is one of the largest structures of the skull, representing approximately three‐fifths of the jaw (Figures 2a–f and S1e, f). Each dentary extends from the most anterior point of the skull to the most posterior point of the maxilla. Its shape is straight and elongated, becoming slightly thicker towards the posterior end, which bifurcates into a long dorsal process and a short ventral process that articulates with the surangular and angular, respectively. Additionally, it articulates dorsoposteriorly with the coronoid via a small dorsal process extending from the long bifurcated process. Posteriorly, it articulates with the splenial along its medial surface. The dentary exhibits small foramina in its anterolateral region for the passage of nerves and blood vessels.

**FIGURE 2:**
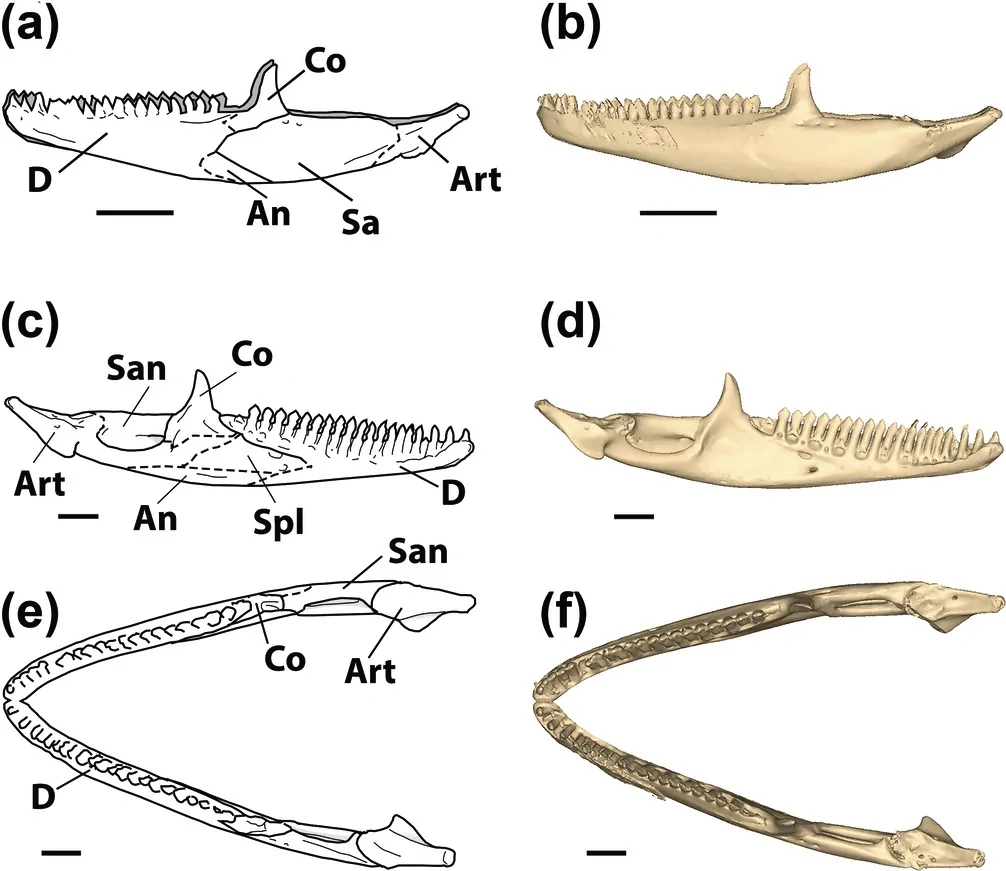
Jaw of the UF:Herp:181922 specimen: (a) drawing of left lateral view, (b) left lateral view, (c) drawing of medial view, (d) medial view, (e) drawing of dorsal view, (f) dorsal view. An, Angular; Art, Articular; Co, Coronoid; D, Dentary; San, Surangular; Spl, Splenial. Dashed lines, inferred sutures based on the literature and other specimens. Scale bars, 1 cm in (a, b), and 0.5 cm in (c, d, e, f).

The coronoid (Co) is a paired bone with a cuneiform shape, prominent in dorsal view, and approximately one‐fifth the height of the skull (Figures 2a–f and S1e, f). It features several processes in its ventral region, resulting in an irregular margin. Anteriorly, it articulates with the dentary and posteriorly with the surangular on its lateral surface. Ventrally, it articulates with the splenial and articular, as well as with the adductor fossa on its medial surface. The adductor fossa is a concave depression present in the medial surface of the jaw formed by some of its bones.

The splenial (Spl) is a paired bone located in the medial view of the jaw, exhibiting a fusiform shape and approximately half the size of the angular bone (Figures 2c, d and S1e, f). It articulates with the dentary anteriorly, with the coronoid dorsally, with the articular posteriorly and with the angular ventrally. The splenial also features a foramen for the passage of the mandibular nerve and blood vessels.

The surangular (San) is a paired bone with an irregular shape, characterized by a narrowing in its medial portion, and measuring approximately one‐fifth the length of the jaw (Figures 2a–f and S1e, f). Its lateral surface articulates with the coronoid via a small anterior process, with the dentary anteriorly through a wide process, with the articular and the quadrate bones dorsoposteriorly, and with the angular ventrally. The surangular articulates with the adductor fossa in its medial surface, with the articular ventrally and with the articular posteriorly. Additionally, it features a foramen in its anterior process, which allows for the passage of nerves and blood vessels associated with the coronoid.

The angular (An) is a paired bone that constitutes nearly one‐fifth of the jaw's length, extending from the ventral region to the medial and lateral aspect of the jaw (Figures 2a–d and S1e–f). It articulates dorsally with the splenial, articular, and surangular, anteriorly with the dentary, and posteriorly with the articular. The angular exhibits a roughly rectangular shape, featuring a thinner anterior region that widens posteriorly with round edges.

The articular (Art) is a paired bone consisting of two distinct regions: an elongated anteromedial, cylindrical portion formed by the fused prearticular, and a posterior region with a thin triangular shape. The articular extends to almost two‐fifths of the total jaw length (Figures 2a–f and S1e–f). The articular is located at the posterior limit of the jaw and exhibits a ventral process that projects medially. On its lateral surface, the articular articulates anteriorly with the surangular, anteroventrally with the angular, and anterodorsally with the quadrate. On the medial surface, the dorsal margin of the articular contributes to the ventral surface of the adductor fossa and articulates anteriorly with the splenial.

### 3.2 Vertebral column

The vertebrae in the vertebral column of *I. iguana* exhibits a range of shapes and sizes based on their anatomical location (Figure 3). The column is divided into four distinct regions: cervical, dorsal, sacral and caudal regions. The only specimen available for this study with a complete skeleton (UF:Herp:181922) exhibited a total of 84 vertebrae.

**FIGURE 3:**
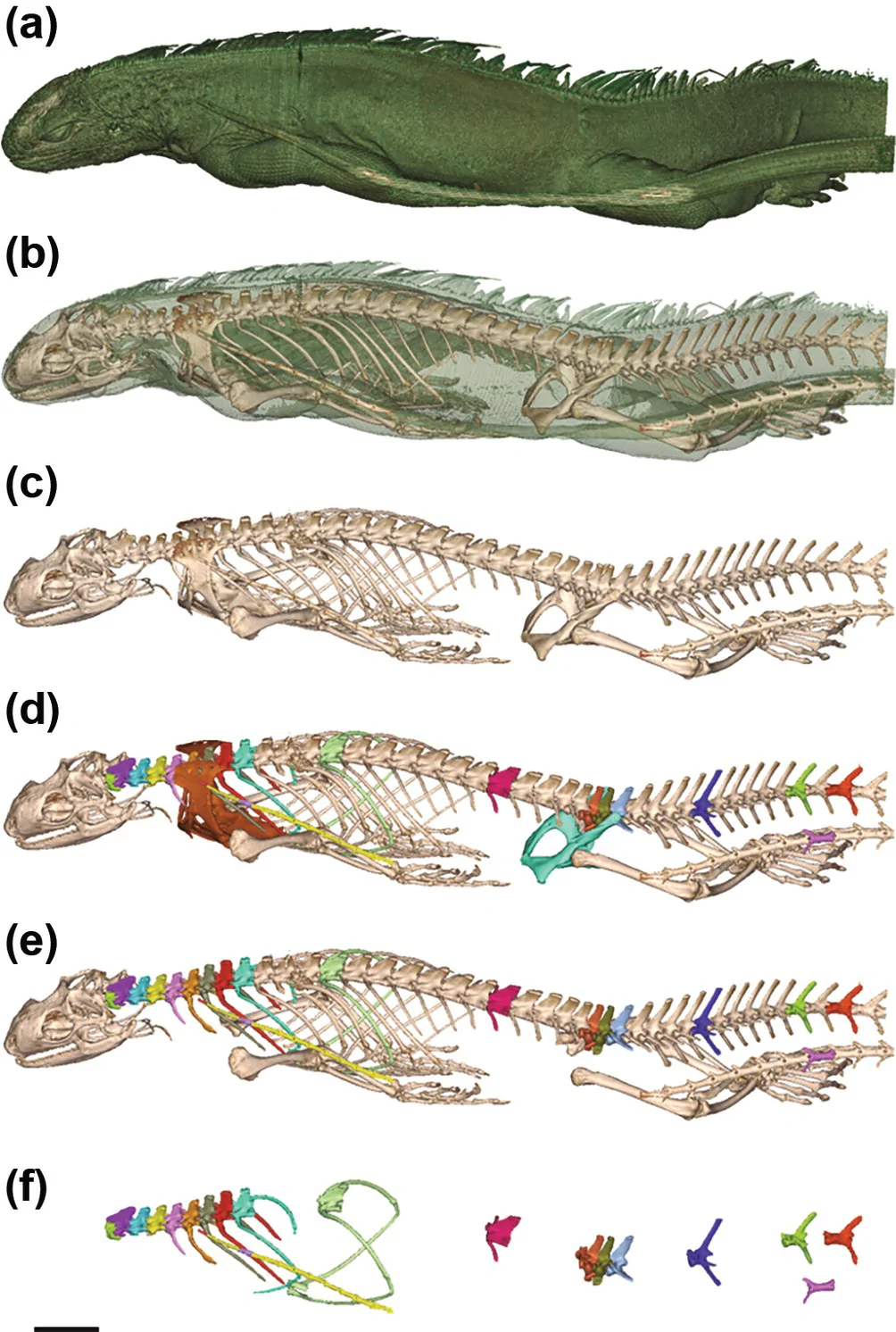
3D reconstruction of *I. iguana* (Ig04) in left lateral view: (a) complete specimen; (b) the same specimen with transparent skin; (c) complete skeleton; (d) skeleton with vertebrae of different segments and girdles colored as to distinguish each structure; (e) skeleton with vertebrae of different segments colored, without the girdles; (f) vertebrae from different segments. Scale bar 5 cm.

### 3.3 Cervical vertebrae

The cervical region (Figures 3c–f, 4 and 5) is defined as the first vertebrae of the vertebral column, which composes the neck, comprising eight vertebrae: the atlas, axis, and the common cervical vertebrae (Cervical 3 to Cervical 8). Hypapophyses are present from the atlas to C5, while cervical ribs are found from C5 to C8 (Figure 4). Both a hypapophysis and a pair of cervical ribs are present in the vertebra C5.

**FIGURE 4:**
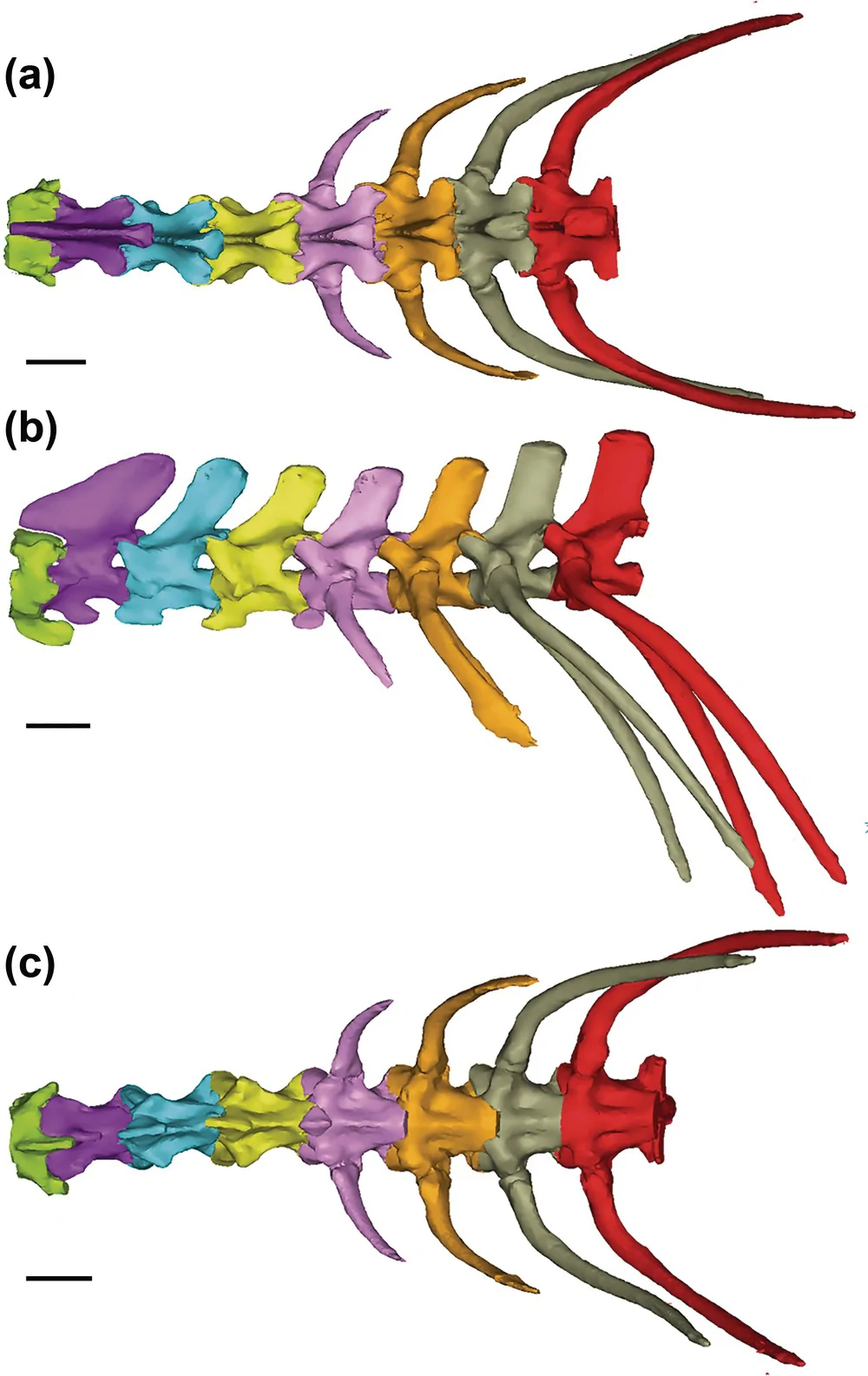
Cervical segment of the Ig04 specimen: (a) dorsal view, (b) left lateral view, (c) ventral view. Colored as to distinguish each structure. Scale bars 1 cm.

**FIGURE 5:**
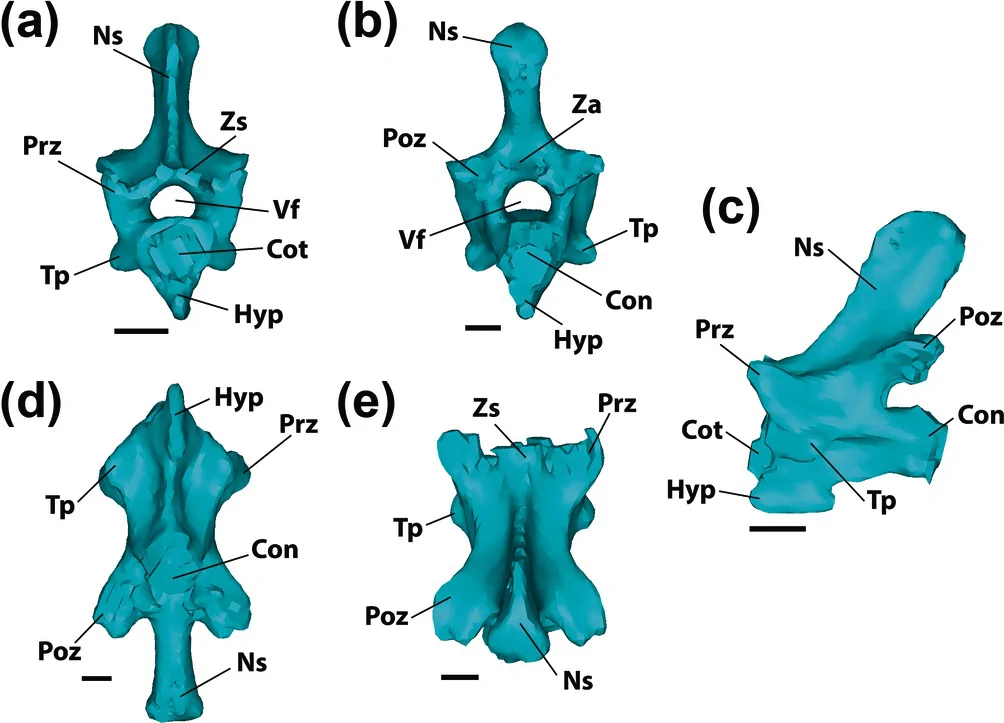
The third vertebra of the cervical segment of the Ig04 specimen: (a) anterior view, (b) posterior view, (c) left lateral view, (d) ventral view, (e) dorsal view. Con, Condyle; Cot, Cotyle; Hyp, Hypapophysis; Ns, Neural spine; Poz, Postzygapophysis; Prz, Prezygapophysis; Tp, Transverse process; Vf, Vertebral foramen; Za, Zygantrum; Zs, Zygosphene. Scale bars, 0.3 cm in (b, d, e), 0.5 cm in (a, c).

The atlas (Figure S3) is the first vertebra of the vertebral column and articulates with the neurocranium via the occipital condyle. It is approximately half the length of the other cervical vertebrae and exhibits a distinctive pentagonal shape. Its vertebral foramen (Vf; Figure S3a–i) occupies nearly the entire vertebra, while the dorsal portion of its vertebral centrum is unfused.

The atlas possesses an anterior transverse process (Tpa) that projects anteriorly from the vertebral centrum to articulate with the occipital condyle (Figure S3a, b, d, e, g, h). This process is triangular and approximately the same size as the prezygapophysis. Additionally, the atlas features a posterior transverse process (Tpp) with a similar triangular shape, though with flattened ends, measuring one‐fourth of the vertebra (Figure S3a–i). This posterior transverse process extends posteriorly towards the axis, articulating with its transverse process. The atlas also features hypapophysis (Hyp), a process extending through its ventral region and projecting posteriorly to nearly one‐third of the axis's length, with a total length twice that of the atlas (Figure S3a–i). However, the atlas lacks both a cotyle (Cot) and a condyle (Con; Figure S3).

The axis, the second vertebra, is notable for possessing the largest neural spine (Ns) in the cervical region, which is oriented anteriorly, but also extends slightly posteriorly (Figure 4a–c). The neural spine is located dorsally to the vertebral centrum and reaches nearly its full length. The shape of the neural spine resembles a triangle with rounded anterior and posterodorsal edges, while the ventral edge is embedded within the vertebral centrum. The axis exhibits a cotyle that supports the odontoid process (Op), which exhibits a cone‐like shape and fits into the vertebral foramen of the atlas anteriorly. Its length matches that of the anterior projection of the neural spine. The cotyle measures approximately one‐fourth the height of the vertebra and expands laterally, presenting a convex surface. The condyle is less developed than the cotyle, measuring about two‐thirds of its size and featuring convex shape.

The vertebrae present prezygapophyses and postzygapophyses that are structured to interlock with adjacent vertebrae (Figure 5a–e). Therefore, the prezygapophyses of the axis are positioned slightly below half the height of the vertebra to articulate anterodorsally with the postzygapophysis of the atlas. The prezygapophyses are cone‐shaped and each accounts for about 1/25 of the total size of the vertebra.

Each postzygapophysis is triangular in shape and measures approximately half the size of the condyle, expanding dorsally towards the neural spine (Figure 5b). A concave depression, known as the zygantrum, is formed between the postzygapophyses at the top of the neural arc (Figure 5b). The zygantrum measures approximately half the size of the vertebral foramen. In contrast, the axis does not articulate with the atlas through a zygosphene, since the atlas does not possess a zygantrum.

The transverse process (Tp) of the axis is located anteriorly on the centrum, below the prezygapophysis, and articulates with the posterior transverse process of the atlas (Figure 4a–c). It is slightly larger than the prezygapophysis and exhibits a similar shape, expanding laterally. The hypapophysis, which comprises one‐fifth of the total height of the vertebra, presents a claw‐like shape with its point oriented posteriorly (Figures 4 and 5a–d).

The centrum of C3 is elongated, similar to the axis's centrum, and this elongation continues in C4, influenced by the increase in the condyle length (Figures 4a–c and 5c–e). However, the centrum progressively decreases in length throughout the cervical region, with the centrum of C8 being approximately one‐seventh shorter than that of the axis (Figure 4a–c). Additionally, the centrum increases in size along the vertebrae, but diminishes in the last two cervical vertebrae. A medial projection extends through the entire ventral length of the vertebrae (Figure 4c). In the first two vertebrae, this projection is thinner and taller, measuring about one‐third of the total centrum height. In subsequent vertebrae, the projection becomes smoother and reduces to one‐fifth of the centrum height. The cotyles possess a concave shape that articulates with the convex condyle of the anterior vertebra, and vice‐versa (Figure 5a–c). Typically, the size of the cotyle is larger than that of the vertebral foramen, while the condyles are equal to or smaller than the foramen.

The neural spine exhibits subtle variations among the cervical vertebrae. All spines possess a rectangular appearance, positioned diagonally in the posterodorsal direction, with an inclined crest oriented anteriorly (Figure 4a, b). The C3 and C4 are positioned highly inclined, featuring a completely curved and diagonal crest. However, from the fifth to the eighth cervical vertebrae, the crest develops a right angle, resulting in a neural spine that is predominantly upright. Additionally, the shape of the neural spine apex varies: cervical vertebrae C3 to C6 exhibit an isosceles triangular shape, while the last two resemble a rectangle with rounded edges. Through these variations, the size of the neural spine remains consistently slightly larger than that of the vertebral centrum.

The hypapophysis is present ventrally in the anterior portion of the vertebrae, being largest in C3 and decreasing progressively until it terminates in C5 (Figure 4a–c). The shapes of the hypapophysis differ among these three vertebrae: in C3, it is elliptical; in C4, it is claw‐shaped and oriented anteriorly; and in C5, it exhibits an oval shape (Figure 4b).

The prezygapophyses are wedge‐shaped with rounded ends, expanding at their anterior portion along the lateral margin of the neural arc to the outer limit of the vertebral centrum (Figure 5a,c,e). The prezygapophysis is relatively small, measuring slightly less than one‐seventh of the vertebra height. The zygosphene, a process located between the prezygapophyses at the top of the neural arc, articulates with the zygantrum of the adjacent vertebra (Figure 5a,e). The zygosphene is wedge‐shaped, but with rounded extremities. In the C3 vertebra, it is enlarged, forming a cohesive structure with the prezygapophysis (Figure 5a). However, from this vertebra onward, the zygosphene becomes progressively thinner and projects more anteriorly, to the point where, after the C5, it no longer contacts the prezygapophysis, creating a fossa between the two processes. The relative size of the zygosphene does not vary significantly, consistently occupying around one‐third of the size of the vertebral foramen.

The postzygapophyses in the C3 resembles that of the axis, however, along the cervical segment it becomes more pronounced (Figure 4a, b and 5b–e). By C6, it assumes a triangular shape that projects dorsolaterally (Figure 4a). In contrast, its ventral portion gradually decreases and turns smoother, outlining the dorsolateral margin of the vertebral foramen (Figure 4a, b). The zygantrum varies in size depending on the shape and size of the postzygapophysis, it can range from nearly the size of vertebral foramen to approximately one‐fourth of it.

The transverse process is in the anterior portion of the lateral surface, extending laterally from the anterior point to approximately one‐third of the length of the centrum (Figure 4a, b). Its height is from the ventral margin of the prezygapophysis to the ventral margin of the cotyle, resulting in a thickened appearance in anterior view, which increases along the cervicals series. The transverse structure gives rise to the pleurapophysis (Ple), a joint surface where the rib (R) articulates (Figures 4a–c and 6a–b). The pleurapophysis and the cervical rib first appear in C4, where they are relatively small (one‐fourth smaller in size than the pleurapophysis of C5), and continue to develop in the subsequent cervical vertebrae, gradually increasing in size. The pleurapophysis originates at C4, located in the ventral one‐third of the vertebral foramen's height; and remains in the same position in C5. In C6, it shifts to the dorsal one‐third of the vertebral foramen's height; while in C7 and C8, it is positioned in the middle of the vertebral foramen. The pleurapophysis extends to the ventral margin of the cotyle, measuring approximately half the height of the vertebra. Its dorsal region begins with a laterally curved projection that extends to the rib joint, which is straight.

**FIGURE 6:**
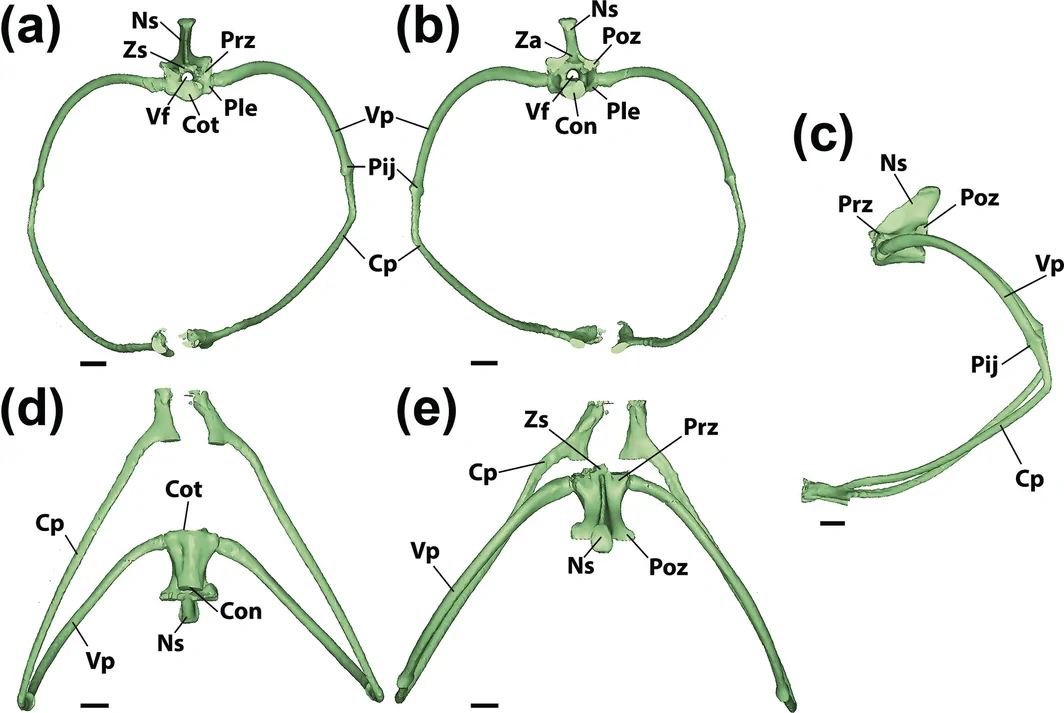
The fifth vertebra of the dorsal segment of the Ig04 specimen: (a) anterior view, (b) posterior view, (c) left lateral view, (d) ventral view, (e) dorsal view. Con, Condyle; Cot, Cotyle; Cp, Cartilaginous portion of the rib; Hyp, Hypapophysis; Ns, Neural spine; Pij, Putative Intracostal Joint; Ple, Pleurapophysis; Poz, Postzygapophysis; Prz, Prezygapophysis; Tp, Transverse process; Vf, Vertebral foramen; Vp, Vertebral portion of the rib; Za, Zygantrum; Zs, Zygosphene. Scale bars 0.8 cm.

The cervical rib exhibits two morphotypes. The first morphotype is present in C5 and C6, displaying similar height and twice the height of the vertebra, respectively (Figure 4a–c). These ribs are curved, with the proximal region nearly matching in height and width of the pleurapophysis, while decreasing in size and flattening as they extend distally. In C5, the distal end narrows, while in C6, it becomes flattened, adopting a rectangular shape. The second morphotype occurs in C7 and C8, resembling those present in the dorsal vertebrae (Figure 4a–c). These ribs are long and cylindrical with narrowed ends. Their size may vary among vertebrae, but are consistent, measuring approximately double the height of the vertebra. A rectangular cartilaginous structure, broader at its midpoint and comprising approximately one‐fifth of the total rib length, was identified near the distal end of the ribs from C5 to C8 (Figure S4).

### 3.4 Dorsal vertebrae

The dorsal region consists of 16 vertebrae, beginning with the first vertebra whose rib articulates with the sternum and concluding with the vertebra that articulates with the first sacral vertebra (Figure 3c–f). The dorsal vertebrae 1, 5 and 12 were selected for analysis to examine variations in the processes within this vertebral segment (Figure S5).

The first dorsal vertebrae exhibit a wide vertebral centrum similar to that of the last cervicals with ribs (Figure 6a–e), gradually increasing in size along the segment until it becomes one‐fifth wider in the last dorsal vertebrae (Figure S5a–i). The length of the centrum also increases along the dorsal vertebrae, with D5 being one‐tenth longer than C8, for example. However, the last three vertebrae are reduced in length, with the last dorsal vertebra being the shortest, measuring two‐sevenths less in length than its anterior vertebra. Additionally, the vertebral centrum undergoes a graduate flattening along the segment. Ventrally, a medial projection forms two lateral fossae that extend for nearly the entire length of the vertebra.

The cotyle and condyle increase in size relative to the cervical vertebrae. In D1, the cotyle is one‐fourth larger than the vertebral foramen; in D5, it measures more than four times the size of the vertebral foramen; and in D12, it exceeds six times the size of the vertebral foramen (Figure S5a–i). The condyle measures approximately twice the size of the vertebral foramen in both D1 and D5, while in D12, it is roughly four times larger. The neural spine is approximately half the total height of the vertebra and shares a similar shape with the last cervical vertebrae (Figure 6a–e). It may exhibit a more diagonal angulation oriented posteriorly or form a right angle oriented dorsally (Figure S5b, e, h). When displaying a diagonal shape, it presents a line angled at approximately 45° in the anterior region, extending to half its length (Figure 6a–e). Posterior to this, the spine curves, forming a right angle of rectangular aspect that is positioned diagonally, featuring an anterior crest. In the vertebrae of the central region of the dorsal segment, the neural spine features a dorsally projected ridge at the midpoint of the anterior surface of its crest (Figure 6a–e).

The shape of the prezygapophyses in the dorsal vertebrae remains similar to that of the last cervical vertebrae, but it features the addition of two lateral projections (Figure 6a–e). In the last dorsal vertebrae, the prezygapophysis loses its pointed appearance, resulting in a smoother contour. Its size is approximately one‐sixth to one‐seventh of the total height and two‐thirds of the total width of the vertebra. Nearly two‐thirds of the initial dorsal vertebrae possess a zygosphene similar to that of the last cervicals. However, in the final third of the segment, the zygosphene becomes smoother and acquires a 90° curvature, with its midpoint oriented dorsally.

The postzygapophyses exhibit a smooth, rounded triangular shape, featuring a dorsal projection that extends to the base of the neural spine (Figure 6b–e). Along with the dorsal segment, the postzygapophyses gradually projects laterally, contributing to an increase in vertebral width. Each dorsal postzygapophysis measures approximately one third of the vertebra's total width. The zygantrum maintains a void appearance, matching the width of the vertebral foramen in the first dorsal vertebrae (Figure 6b). It gradually expands laterally, eventually adding approximately one‐third of the vertebral foramen's width on each side (Figure S5c, f, i). In terms of height, the zygantrum is typically equal to that of the vertebral foramen, with an increase of one‐third observed in the last dorsal vertebrae.

The pleurapophysis is characterized as a laterally rounded projection that extends from the height of the dorsal margin of the vertebral foramen to the height of the ventral margin of the cotyle, constituting approximately one‐third of the vertebra's height (Figure 6a, b). The pleurapophysis articulates with the rib via the costovertebral joint, characterized by a rounded, laterally projected single‐head structure.

The first six dorsal vertebrae possess long sternal ribs that form the rib cage (Figure 7). The anterior four vertebrae feature direct sternal ribs that articulate directly with the sternum bone of the pectoral girdle. In contrast, the subsequent two vertebrae feature indirect sternal ribs that fuse distally, creating a cylindrical and flattened cartilaginous structure with a “Y” shape that articulates with the sternum at its posterior region. The length of the first sternal rib is approximately six times the width of its corresponding vertebra, gradually increasing to nine times the width by the last sternal rib. These ribs are divided into two segments: a proximal ossified vertebral portion (Vp) and a distal cartilaginous sternal portion (Cp), which articulate through the putative intracostal joint (Pij), also known as “elbow” (Figure 6a–e). Ventral to the costovertebral joint, there is a slightly rounded projection, followed by a posterior curvature. The distal extremity of the cartilaginous segment is rounded or may exhibit a plateau shape, where it articulates with the sternum.

**FIGURE 7:**
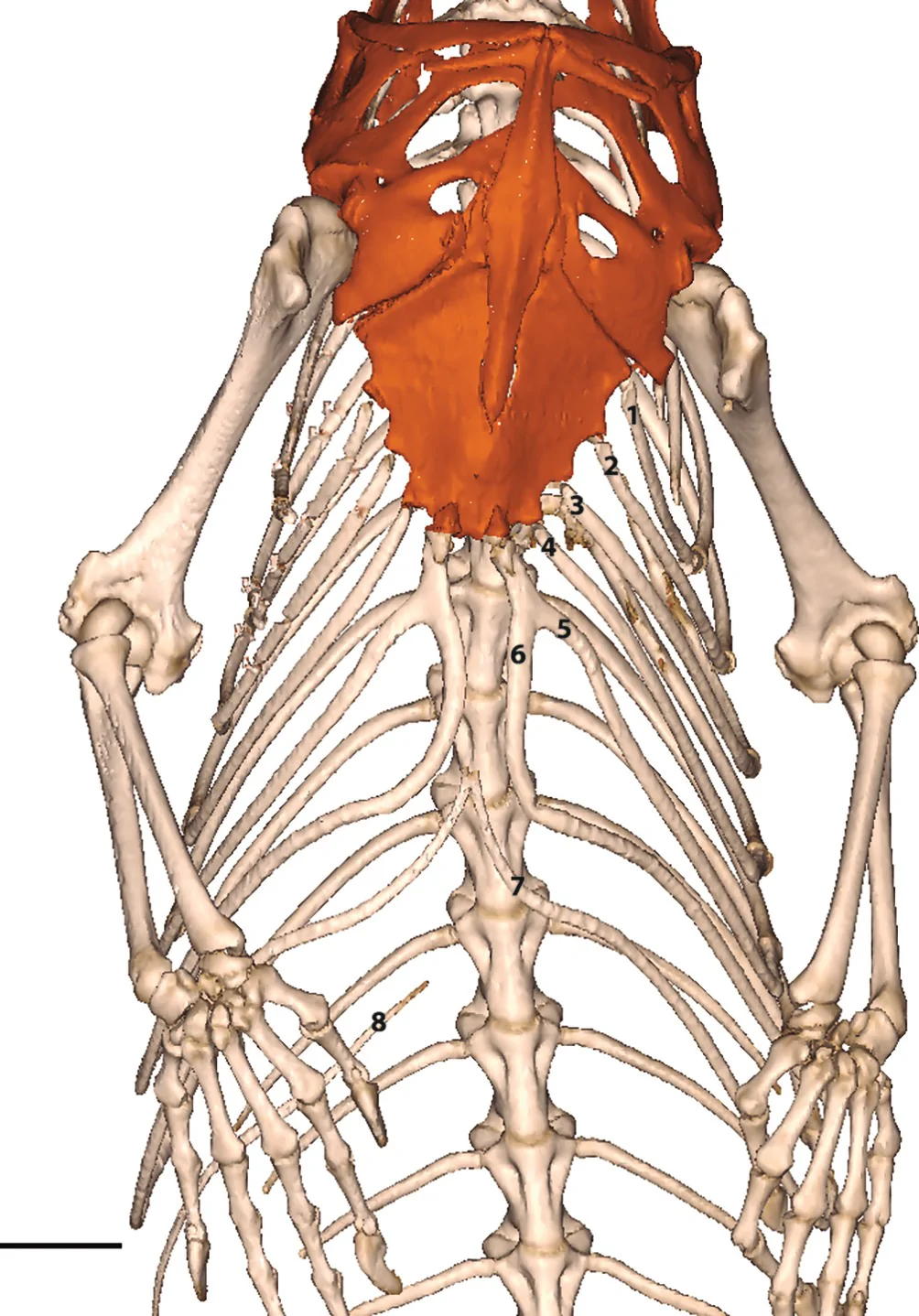
Rib cage of the Ig04 specimen in ventral view: (1–4) direct sternal ribs, (5–6) indirect sternal ribs, (7–8) long vertebral ribs. Brown, pectoral girdle. Scale bar 2.5 cm.

The vertebral ribs are present in the last 10 vertebrae of the dorsal segment. The first two vertebrae, D7 and D8, exhibit long free ribs that are similar in size to the preceding ribs, measuring approximately eight times and seven times the width of their respective vertebrae (Figure 7). The D7 and D8 nearly connect distally at both ends of its cartilaginous extremities, almost forming a “V” shape. The vertebrae maintain the rib morphology of both vertebral and cartilaginous segments, featuring the “elbow” joint. Following these, there are eight small free‐rib vertebrae that lack the cartilaginous segment, retaining only a small vertebral segment (Figure S5g–i). These ribs decrease in size towards the last dorsal vertebra, becoming shorter and more robust (Figure 3c–f). The ribs of the D9 are approximately four times the width of their vertebra, while the last dorsal vertebrae possess ribs that are slightly wider than the vertebra itself. As observed in D12, the last dorsal vertebrae exhibit short and straight ribs that narrow to rounded extremities (Figure S5g–i).

### 3.5 Sacral vertebrae

The sacral region consists of two vertebrae (Sa1 and Sa2) that articulate laterally with the pelvic girdle (Figure S6). The vertebral centrum is reduced and slender compared to the vertebrae in other segments, measuring approximately three‐fourth the length of the last dorsal vertebra's centrum. In Sa1, the cotyle and condyle are nearly twice the size of the vertebral foramen (Figure S6a, c). In contrast, in Sa2, although larger than the vertebral foramen, these structures do not reach twice its size (Figure S6d, f). The vertebral foramen in Sa1 is triangular, whereas in Sa2, it is oval‐shaped.

The neural spine is rectangular and slender, measuring approximately three‐fifths of the total vertebral height, contributing to an increase of one‐sixth in overall vertebra size (Figure S6b, e). The prezygapophyses of the first sacral vertebra has a triangular shape, accounting for nearly one‐fifth of the total width of the vertebra (Figure S6a). Its dorsal diagonal margin forms a crest that extends medially from the ventral margin of the vertebral foramen to the same height as the zygosphene laterally. In the second sacral vertebra, the prezygapophyses decrease in size to one‐seventh of the total width each (Figure S6d). Consequently, its morphology becomes slenderer, with the extremities resembling dorsally oriented spikes.

The zygosphene of Sa1 maintains a similar shape to the final dorsal vertebrae, projecting anteriorly as two slightly rounded, flattened structures (Figure S6a). In Sa2, both structures of the zygosphene become more linear and diagonal, creating an arrow‐like appearance as they meet medially at a peak (Figure S6d). The zygosphene is slightly smaller than the prezygapophysis.

The postzygapophysis retains its triangular shape, constituting approximately one‐eighth of the total width of the vertebra (Figure S6c, f). The zygantrum maintains its concavity without significant changes in shape (Figure S6c, f). In Sa1, its size is slightly smaller than that of one postzygapophysis, whereas in Sa2, it increases in size, reaching the same size as the postzygapophysis and becoming deeper.

The pleurapophysis is the main distinguishing feature of these vertebrae compared to other segments, forming a long, wide lateral rectangular projection with enlarged dorsal and ventral distal regions that articulate with the pelvic girdle (Figure S6a–g). In Sa1, the pleurapophysis is smooth, measuring nearly one‐fourth of the vertebra's width and one‐third of its height. In Sa2, it develops a crest along its anterior surface, starting laterally at the distal region and extending medially to the beginning of the vertebral centrum, where it articulates with Sa1. The pleurapophysis of Sa2 measures approximately three‐sevenths of the total vertebral width, and a foramen is present on both its anterior and posterior surfaces.

### 3.6 Caudal vertebrae

The final region of the vertebral column, the caudal vertebrae, consists of all vertebrae posterior to the sacrals, extending to the last vertebrae of the organism. It represents the largest vertebral segment, and due to its size and function, seven distinct morphotypes have been identified along its length in this study (Figures 8, 9, 10, 11, 12, 13, 14, 15). While the presence of these morphotypes varies among individuals, their sequential order remains consistent, from one to seven. The number of vertebrae in each morphotype will be assessed later. In this section, we focus on describing each morphotype, regardless of the specimens in which they were found.

**FIGURE 8:**
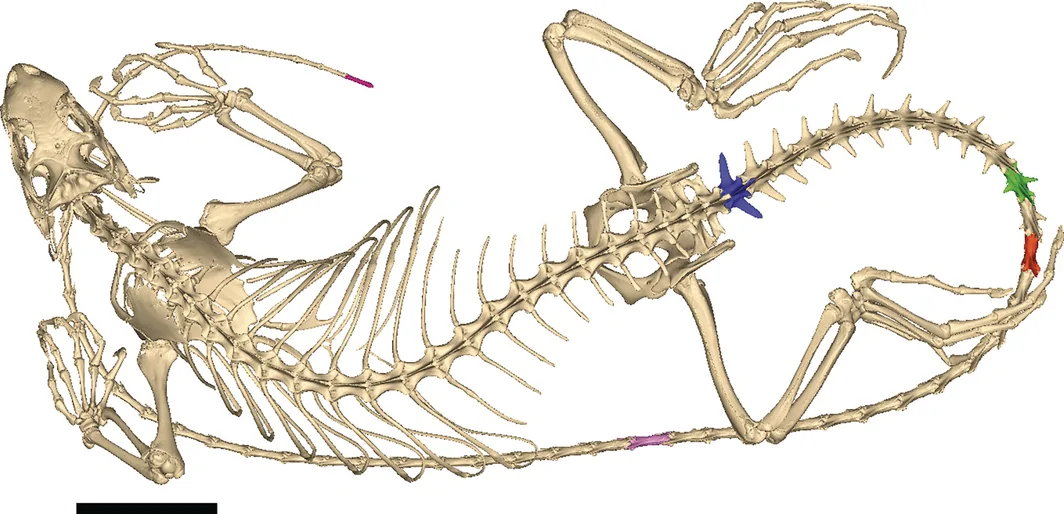
Skeleton of the UF:Herp:181922 specimen with its caudal vertebrae morphotypes 2, 3, 4, 5, and 6, highlighted. Scale bar 5 cm.

**FIGURE 9:**
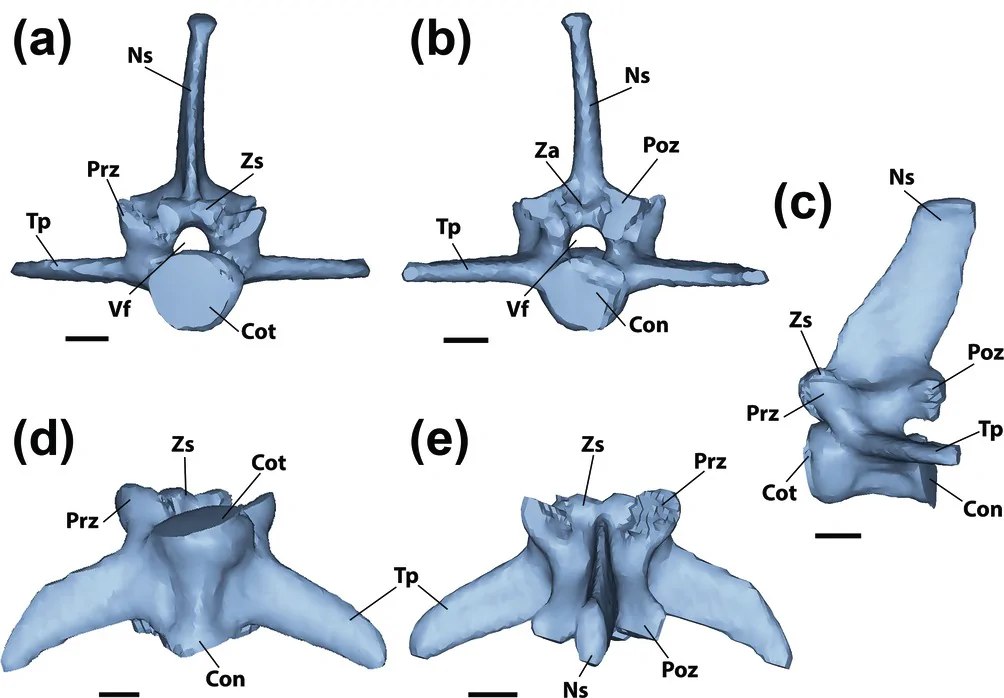
Morphotype 1 of the caudal vertebrae of the Ig04 specimen: (a) anterior view, (b) posterior view, (c) left lateral view, (d) ventral view, (e) dorsal view. Con, Condyle; Cot, Cotyle; Ns, Neural spine; Poz, Postzygapophysis; Prz, Prezygapophysis; Tp, Transverse process; Vf, Vertebral Foramen; Za, Zygantrum; Zs, Zygosphene. Scale bars, 0.5 cm in (a, b, c, e) and 0.4 cm in (d).

**FIGURE 10:**
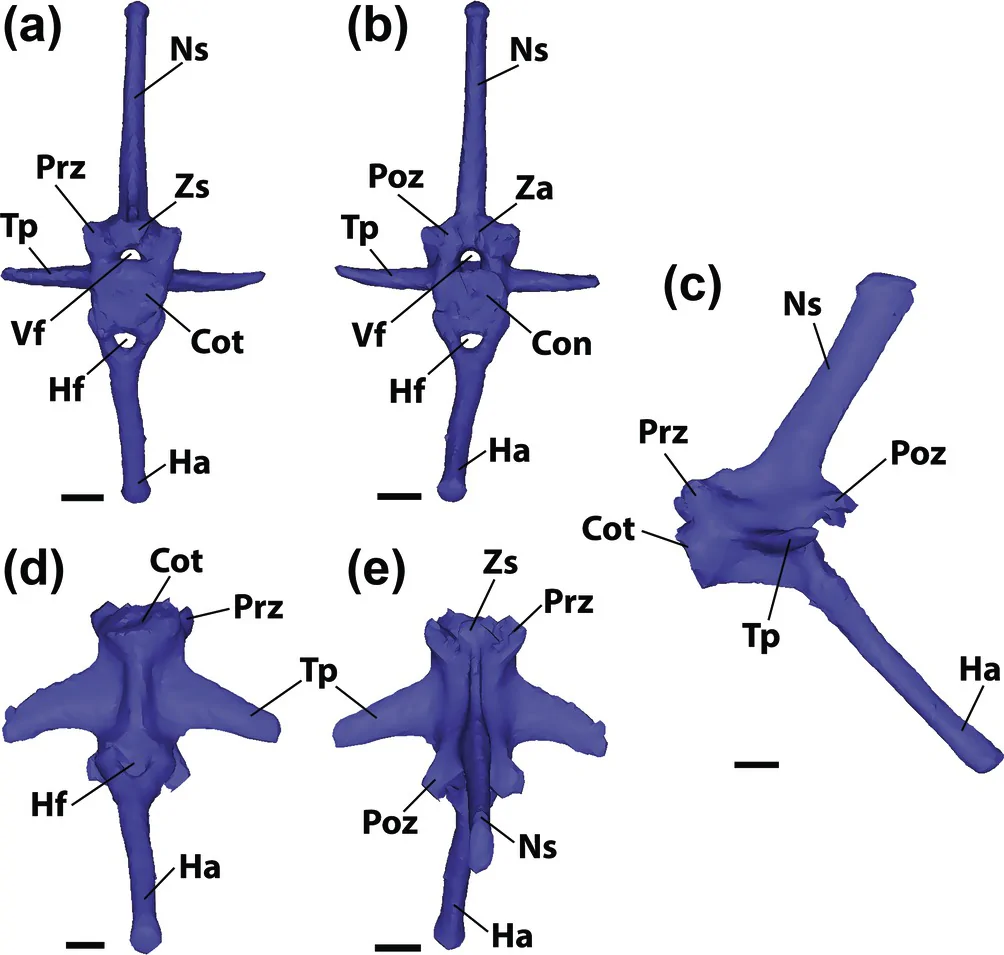
Morphotype 2 of the caudal vertebrae of the Ig04 specimen: (a) anterior view, (b) posterior view, (c) left lateral view, (d) ventral view, (e) dorsal view. Con, Condyle; Cot, Cotyle; Ha, Haemal arch; Hf, Haemal foramen; Ns, Neural spine; Poz, Postzygapophysis; Prz, Prezygapophysis; Tp, Transverse process; Vf, Vertebral Foramen; Za, Zygantrum; Zs, Zygosphene. Scale bars, 0.5 cm in (a, b, c, e) and 0.4 cm in (d).

**FIGURE 11:**
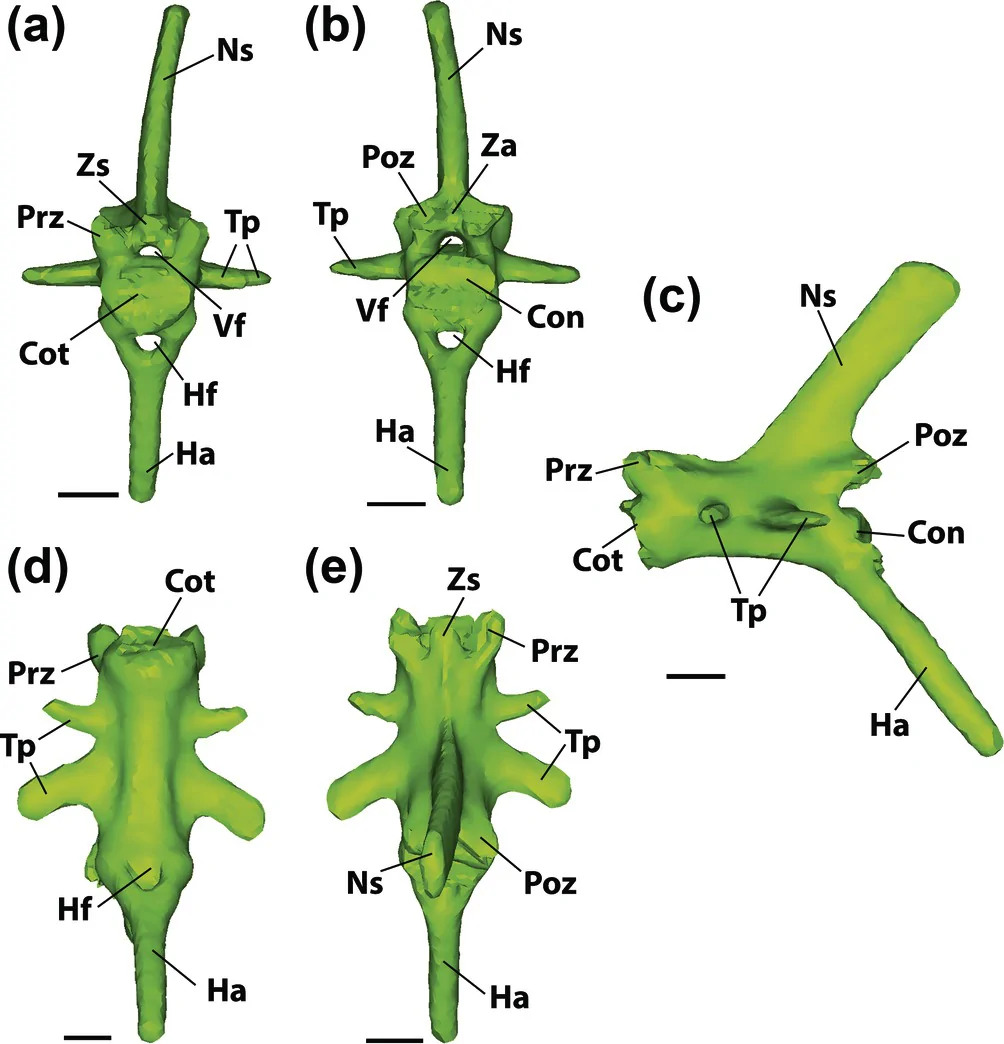
Morphotype 3 of the caudal vertebrae of the Ig04 specimen: (a) anterior view, (b) posterior view, (c) left lateral view, (d) ventral view, (e) dorsal view. Con, Condyle; Cot, Cotyle; Ha, Haemal arch; Hf, Haemal foramen; Ns, Neural spine; Poz, Postzygapophysis; Prz, Prezygapophysis; Tp, Transverse process; Vf, Vertebral Foramen; Za, Zygantrum; Zs, Zygosphene. Scale bars, 0.5 cm in (a, b, c, e) and 0.4 cm in (d).

**FIGURE 12:**
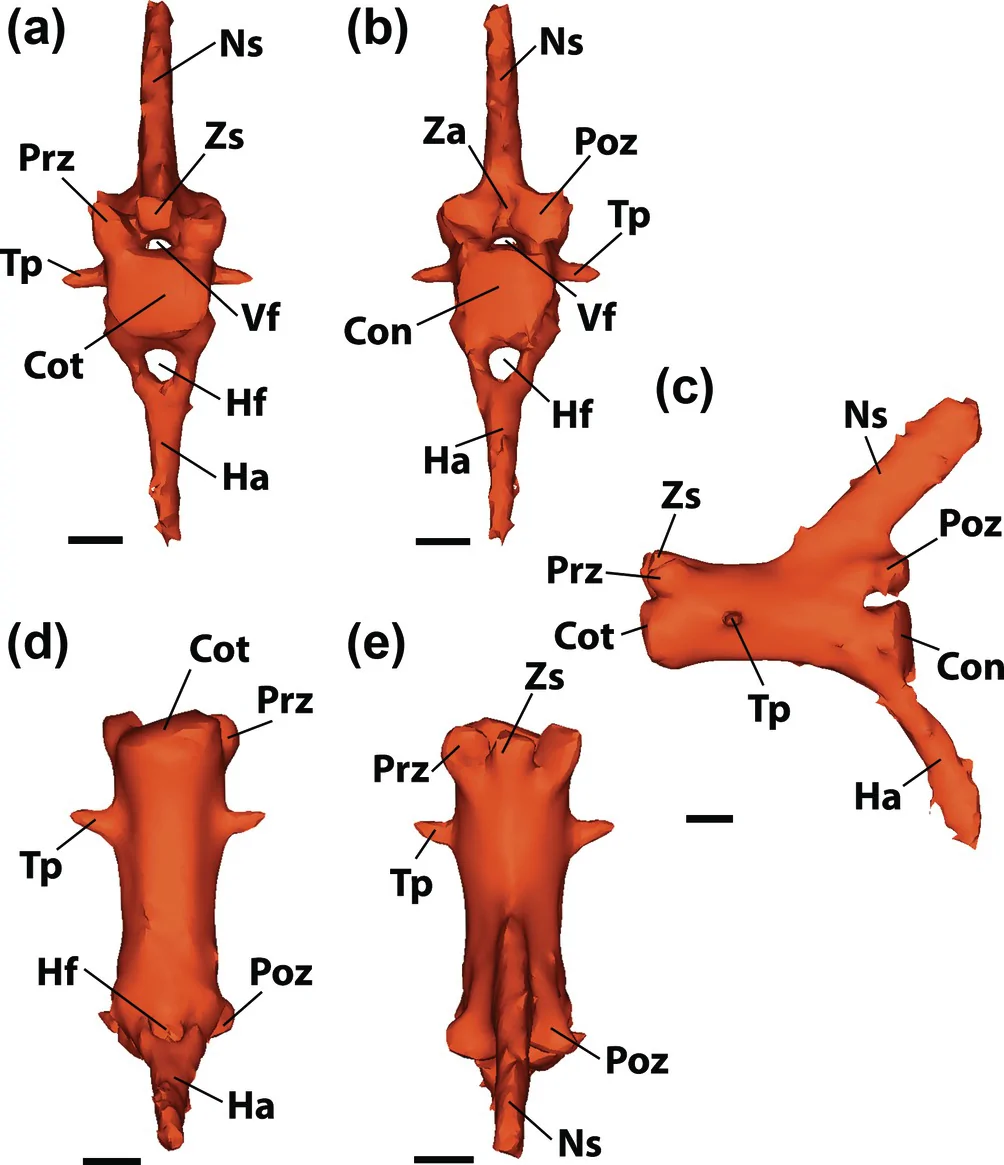
Morphotype 4 of the caudal vertebrae of the Ig04 specimen: (a) anterior view, (b) posterior view, (c) left lateral view, (d) ventral view, (e) dorsal view. Con, Condyle; Cot, Cotyle; Ha, Haemal arch; Hf, Haemal foramen; Ns, Neural spine; Poz, Postzygapophysis; Prz, Prezygapophysis; Tp, Transverse process; Vf, Vertebral Foramen; Za, Zygantrum; Zs, Zygosphene. Scale bars 0.4 cm.

**FIGURE 13:**
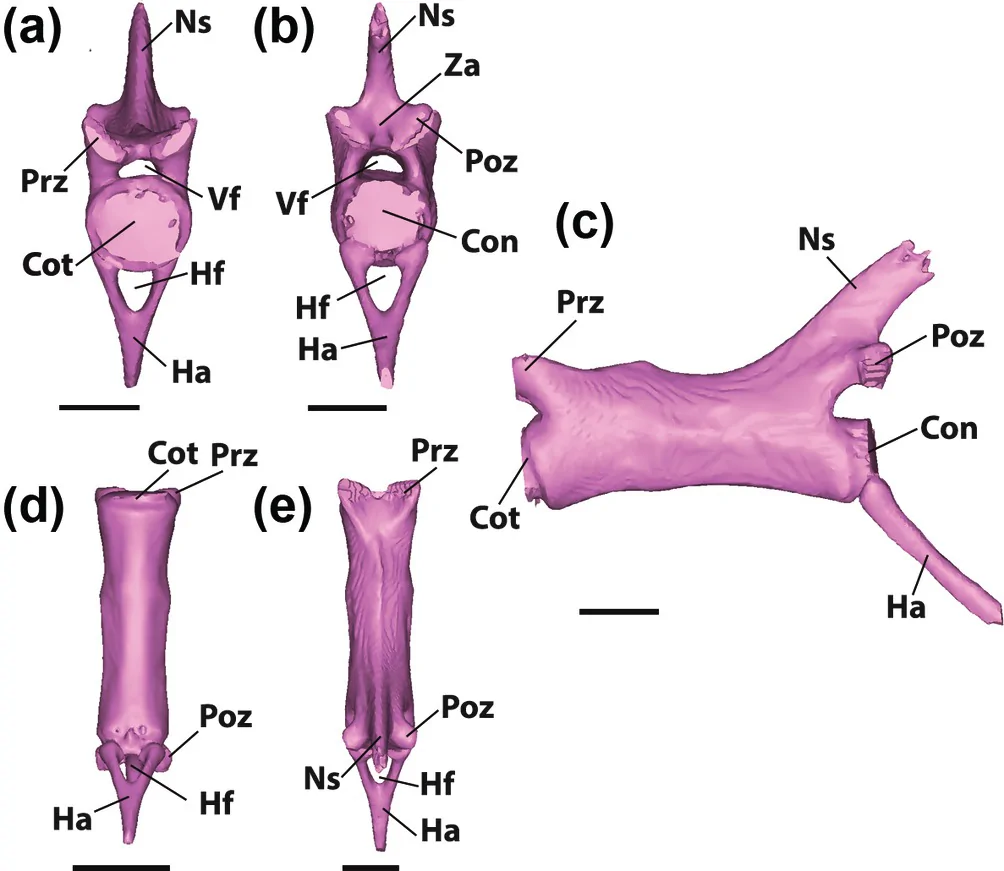
Morphotype 5 of the caudal vertebrae of the UF:Herp:181922 specimen: (a) anterior view, (b) posterior view, (c) left lateral view, (d) ventral view, (e) dorsal view. Con, Condyle; Cot, Cotyle; Ha, Haemal arch; Hf, Haemal foramen; Ns, Neural spine; Poz, Postzygapophysis; Prz, Prezygapophysis; Vf, Vertebral Foramen; Za, Zygantrum. Scale bars, 0.3 cm in (a, b, c, e) and 0.5 cm in (d).

**FIGURE 14:**
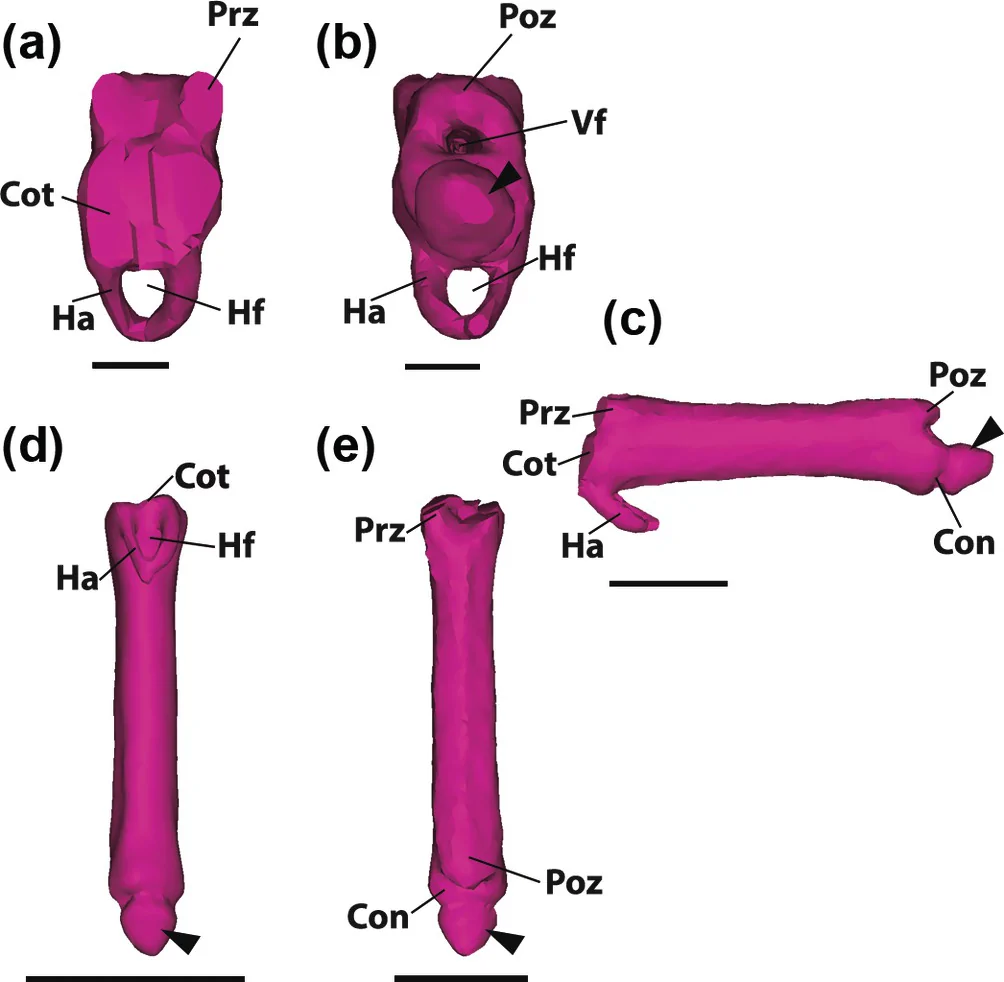
Morphotype 6 of the caudal vertebrae of the UF:Herp:181922 specimen: (a) anterior view, (b) posterior view, (c) left lateral view, (d) ventral view, (e) dorsal view. Con, Condyle; Cot, Cotyle; Ha, Haemal arch; Hf, Haemal foramen; Poz, Postzygapophysis; Prz, Prezygapophysis; Vf, Vertebral Foramen. The triangle indicates the cone‐like structure observed only in this specimen. Scale bars, 0.1 cm in (a, b), 0.3 cm (c, e), and 0.5 cm in (d).

**FIGURE 15:**
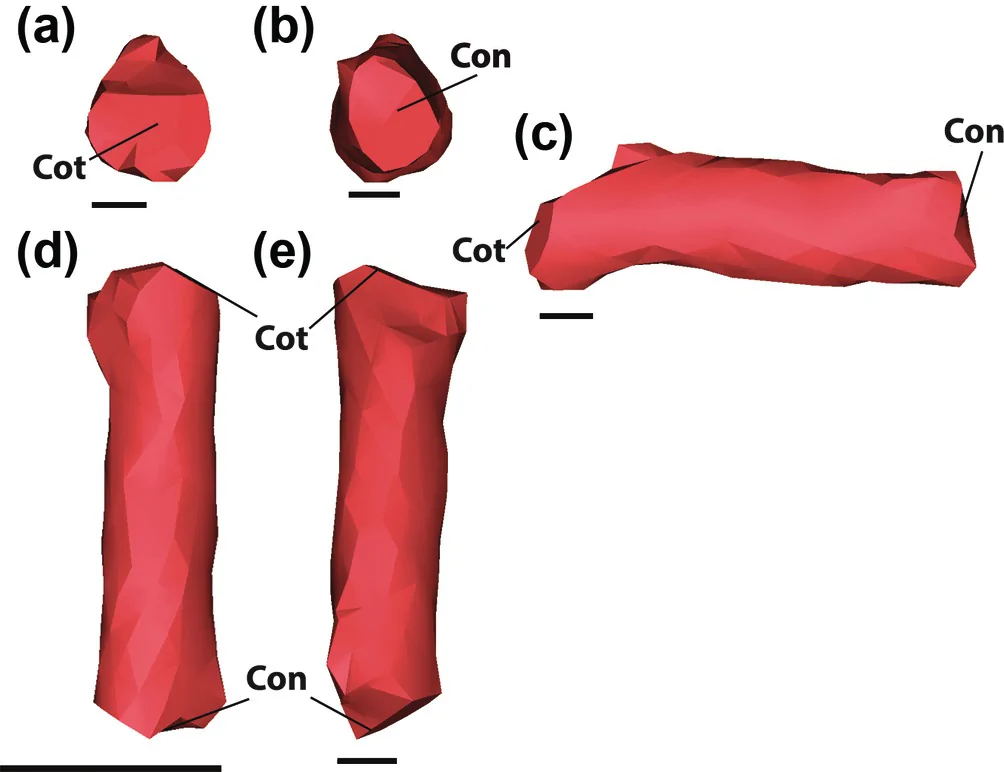
Morphotype 7 of the caudal vertebrae of the Ig04 specimen: (a) anterior view, (b) posterior view, (c) left lateral view, (d) ventral view, (e) dorsal view. Con, Condyle; Cot, Cotyle. Scale bars, 0.1 cm in (a, b, c, e) and 0.4 cm (d).

#### 3.6.1 Morphotype 1

The morphotype 1 (Figure 9), located on the proximal region of the tail, closely resembles the sacral vertebrae due to its wide, flattened vertebral centrum, although the vertebrae of morphotype 1 exhibits a slight decrease in height and increase in width. The cotyle is approximately two to three times the size of the vertebral foramen, while the condyle is approximately three times its size (Figure 9a, b). The neural spine is thin, relatively triangular, and posteriorly oriented, with its height slightly exceeding the midpoint of the total height of the vertebra (Figure 9c,e).

The prezygapophyses expand laterally in a triangular shape with rounded edges, composing one‐seventh of the total width of the vertebra (Figure 9a). The zygosphene appears with a rounded shape, matching the width of the prezygapophysis and being nearly the same height as the vertebral foramen (Figure 9a). The zygosphene is divided into two parts, similar to the sacrals, forming a fossa between them (Figure 9b). The postzygapophysis retains its lateral triangular shape as seen in the sacral vertebrae, measuring around one‐eighth of the vertebra's total width (Figure 9b). The zygantrum retains its typical shape but is deeper than in the preceding vertebrae, measuring approximately one‐seventh of the vertebra's total width (Figure 9b).

A distinctive feature of this morphotype is its transverse process shape, which remains as wide as the sacral pleurapophysis, constituting one‐third of the total width (Figure 9a–e). It is a thin structure that narrows towards its distal point, which is strongly posteriorly oriented, extending slightly beyond the condyle.

The haemal arch (Ha) is a straight ventral projection of the vertebral centrum posteriorly oriented (Figure 10a–e). Its dorsalmost contacts with the centrum in a “Y”‐shaped structure, forming the space for the haemal foramen (Hf). The distal extremity of the haemal arch extends posteriorly. Although the morphotype 1 typically lacks a fully developed haemal arch, vertebrae displaying an underdeveloped haemal arch, representing a transition between morphotype 1 and 2, were classified as part of this morphotype. This morphotype was found in the Ig01 and Ig04 specimens.

#### 3.6.2 Morphotype 2

The morphotype 2 (Figure 10) is positioned in the proximal region of the tail, and its shape differs drastically from morphotype 1, being more slender, elongated, and thin. Its first vertebrae are the largest in the caudal segment, but progressively decreases in size, with the processes reducing proportionally, and elongates as the series extends. The vertebral centrum is thinner and more elongated compared to morphotype 1 (Figure 10a–e). The cotyle is approximately three times the size of the vertebral foramen, while the condyle is about twice its size (Figure 10a). The neural spine is thin and pronounced, accounting for half the total height of the vertebra (Figure 10a–c, e). It transitions from a shorter, triangular shape to an elongated, thinner, rectangular form, extending posteriorly beyond the condyle to about half the length of the vertebral centrum. As the series continues, the neural spine becomes increasingly inclined in the posterior orientation (Figure 3c–f). The pseudospine crest is also present, constituted by a small dorsal protuberance located in the dorsal region of the centrum, anterior to the neural spine, and at its maximum reaches one‐fourth of the vertebral centrum's height.

The prezygapophyses retain the same shape as morphotype 1, but decrease in width to one‐sixth of the total vertebral width (Figure 10a,c–e). The zygosphene also reduces in size, becoming one‐fourth of the width of the vertebral centrum (Figure 10a). The postzygapophysis maintains its shape, but is positioned slightly more dorsally, with a width slightly less than half that of the vertebral centrum (Figure 10b). Consequently, the zygantrum measures approximately one‐fourth the width of the centrum (Figure 10b).

The transverse process is long, constituting approximately 1/3 of the total vertebral width, tapering along its length and oriented posteriorly (Figure 10a–e). Throughout this section of the tail, the transverse processes gradually decrease in size, becoming more slender and increasingly posteriorly oriented (Figure 3c–f). The haemal arch measures roughly one‐third of the vertebral height and extends backwards from the condyle, measuring slightly more than the length of the neural spine (Figure 10a–e). As the vertebrae decrease in size along the segment, the haemal arch length also reduces, potentially reaching half the vertebra's length. The morphotype 2 was identified in the Ig01, Ig04 and UF:Herp:181922 specimens.

#### 3.6.3 Morphotype 3

The morphotype 3 (Figure 11) occurs in the transitional region of the tail and resembles morphotype 2, however, it features an additional transverse process located anterior to the original one. This anterior process is smaller, measuring approximately two‐thirds the length of the posterior transverse process, which itself is three‐fourth the width of the vertebral centrum (Figure 11c–e). The two processes diverge distally: the anterior process is oriented anteriorly, while the posterior process is oriented posteriorly. The cotyle and condyle are nearly four times the size of the vertebral foramen (Figure 11a, b). The neural spine extends posteriorly beyond one‐fourth of the length of the vertebral centrum, while the haemal arch extends beyond one‐half of the length of the centrum (Figure 11a–e). This morphotype occurs in the Ig04 and UF:Herp:181922 specimens.

#### 3.6.4 Morphotype 4

The morphotype 4 (Figure 12) is located in the transitional region of the tail and follows the morphology seen in morphotype 3, except for the absence of the posterior transverse process and in being slightly smaller in size. This anterior transverse process measures approximately one‐fifth of the width of the vertebral centrum and is oriented slightly anteriorly (Figure 12a–e). The neural spine continues to project posteriorly to the condyle, measuring one‐fourth the length of the vertebral centrum, while the haemal arch reduces in size, becoming the same size as the neural spine (Figure 12a–e). The prezygapophysis and the zygosphene each present one‐third of the width of the centrum, with their shape becoming more rounded and flattened (Figure 12a,c–e). The postzygapophysis presents approximately half of the width of the vertebral centrum, accompanied by a thin zygantrum of one‐tenth the width of the centrum (Figure 12b–e). This morphotype is found in the Ig04 and UF:Herp:181922 specimens.

#### 3.6.5 Morphotype 5

The morphotype 5 (Figure 13) originates in the transitional region and may extend into the distal region of the tail. It is characterized by the absence of transverse process, and the presence of the neural spine and the haemal arch (Figure 13a–e). Its vertebral centrum is elongated and thin, with the vertebral foramen decreasing to one‐fifth the width of the vertebral centrum (Figure 13a–e). The cotyle and condyle are five times larger than the size of vertebral foramen (Figure 13a, b). The neural spine curves posteriorly, resembling a hook, and its size decreases, projecting only one‐seventh of the centrum's length posterior to the condyle (Figure 13a–c, e). Additionally, it is located more posteriorly relative to the preceding vertebrae, constituting one‐fourth of the length of the posterior region of the vertebral centrum. The haemal arch also reduces in length and projects posteriorly, equal to the neural spine (Figure 13a–e). Consequently, the haemal foramen becomes proportionally larger, measuring twice the size of the vertebral foramen and one‐fourth the length of the haemal arch.

The prezygapophyses are oval structures anterodorsally oriented, measuring one‐fifth the width of the vertebra (Figure 13a). They lack a zygosphene, instead presenting only a fossa that is two‐fifths the vertebra's width (Figure 13a). The postzygapophysis narrows dorsoposteriorly, constituting three‐fifths the width of the condyle (Figure 13b). The zygantrum resembles a depression line, measuring one‐fifth of the width of the centrum (Figure 13b). This morphotype is present in the Ig01, Ig04, and UF:Herp:181922 specimens.

#### 3.6.6 Morphotype 6

The morphotype 6 (Figure 14) was identified exclusively in the UF:Herp:181922 specimen and constitutes its final vertebral series, spanning from Ca43 to Ca58, comprising a total of 16 vertebrae. Therefore, is positioned in the distal region of the tail. This morphotype exhibits only the haemal arch, lacking transverse processes, neural spine, zygosphene and zygantrum (Figure 14a–e). Its vertebral centrum is elongated and slender. The prezygapophyses are oval‐shaped and are projected anteriorly, reaching approximately one‐third the height of the vertebra (Figure 14a). The postzygapophysis fuses medially, forming an oval structure that extends posteriorly to about one‐fourth the height of the vertebra (Figure 14b).

The haemal arch decreases in length, reaching one‐fifth the length of the vertebra (Figure 14a–e). Its shape transforms into two cylindrical structures that form the haemal foramen, losing the previous straight ventral line. Consequently, the haemal foramen reduces in size, occupying slightly less than one‐third of the total height of the vertebra. The haemal arch position also changes, initially, it articulates with the ventroposterior region of the centrum. However, after the Ca45, it articulates with the anterior region of the vertebral centrum of the immediately posterior vertebra. By Ca50, the haemal arch is identified in the ventroanterior region of the vertebral centrum, a position that is maintained in the final eight caudal vertebrae. The last vertebra, Ca58, features a cone‐like structure that measures one‐ninth the length of the vertebra, which articulates with the condyle posteriorly (Figure 14b–e).

#### 3.6.7 Morphotype 7

The morphotype 7 (Figure 15) is identified solely in the Ig04 specimen, consisting of the last 14 vertebrae and corresponding to the distal region of the tail. Due to a rupture in the tail preceding these vertebrae, the exact number of vertebrae cannot be determined. This morphotype is characterized by a vertebra without processes and a neural spine, featuring a thin and elongated centrum (Figure 15a, e). Its length is less than half that of the vertebrae in morphotype 5 and progressively decreases throughout the rest of the segment. It lacks prezygapophysis, postzygapophysis, as well as zygosphene and zygantrum.

### 3.7 Pectoral girdle

The suprascapula (Ss; Figure 16) is the most dorsal element of the pectoral girdle and is positioned dorsally to the last cervical vertebrae. It is a paired blade with a fan‐like shape, characterized by a broader dorsal portion and narrower ventral portion, where it articulates with the scapula. Anterior to this articulation, both the suprascapula and scapula also articulate with the clavicle. The suprascapula is similar in size to the scapula, but slightly wider. The scapula (Sc; Figure 16) is a paired bone that resembles a trapezoid shape with two fenestras in its anterior region: one dorsal and the other ventral. It is nearly half the size of the procoracoid, in which it articulates ventrally and shares its ventral fenestra.

**FIGURE 16:**
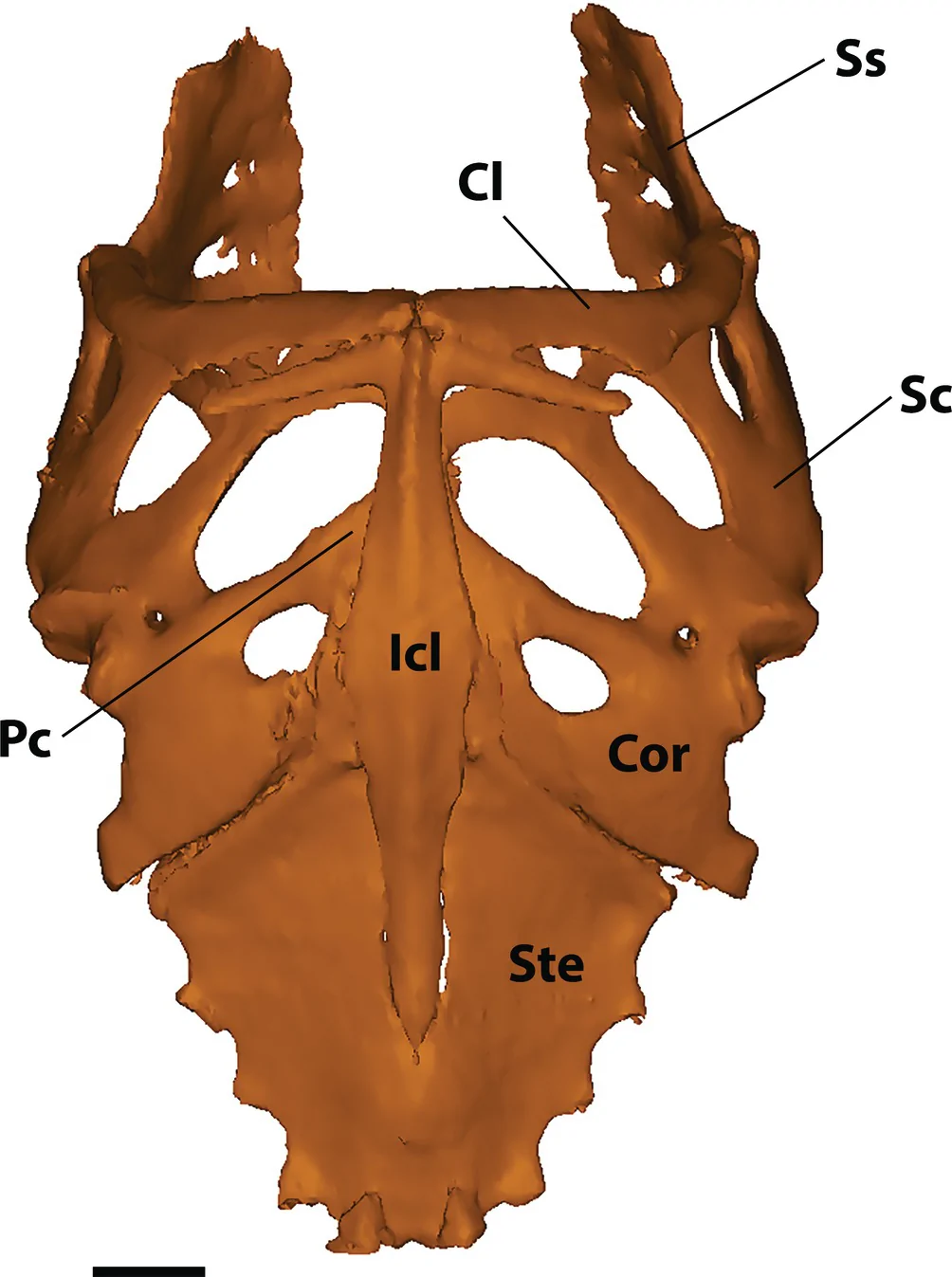
Pectoral girdle of the Ig04 specimen and sternum in ventral view. Cl, Clavicle; Cor, Coracoid; Icl, Interclavicle; Pc, Procoracoid; Sc, Scapula; Ss, Suprascapula; Ste., Sternum. Scale bar 1 cm.

The procoracoid (Pc; Figure 16) is a slender paired bone that measures approximately one‐sixth the width of the coracoid. The procoracoid is positioned internally relative to the interclavicle. At its anterior margin, it exhibits a straight shape, while at its lateroposterior margin it is irregular, characterized by three concave shapes. These concavities form the medial and anterior margins of the three foramina present in the coracoid and the foramen of the scapula, thus articulating with these two bones. Medially, the procoracoid forms a semicircular shape that overlaps with its counterpart.

The coracoid (Cor; Figure 16) is a paired bone with an irregular, wide and convex shape, measuring half the total width of the pectoral girdle and featuring four openings. There are three fenestrae: a dorsal one that is shared with the scapula, a medial one located in its central and a ventral one. They measure, respectively, one‐third, two‐thirds and one‐fourth of the total width of the procoracoid. There is also a small foramen, about one‐twentieth of the width of the procoracoid, known as the coracoid foramen. Ventrolateral to the articulation between the procoracoid and scapula bones, lies an articular surface for the humerus. The procoracoid articulates laterodorsally with the scapula and ventrally with the sternum. At its ventral margin, a small process is located laterally.

The clavicle (Cl; Figure 16) is a slender, straight, paired bone located at the anterior region of the pectoral girdle, measuring nearly half its width. The clavicle articulates laterally with the scapula and suprascapula, while medially with its counterpart and interclavicle. The interclavicle (Icl; Figure 16) is an arrow‐shaped bone oriented anteriorly, that widens at its central portion and tapers posteriorly. Positioned centrally within the pectoral girdle, it measures approximately three‐fourths of the length of its ventral facet. The interclavicle articulates anteriorly with the clavicles at their medial connection and ventrally with the sternum. The most posterior point of the interclavicle is surrounded by a slender foramen.

The sternum (Ste; Figure 16) is a diamond‐shaped bone located in the posterior region of the pectoral girdle, with about half of the length. It articulates anterolaterally with the procoracoid and medially with the interclavicle at its central region. Ten semicircular articular surfaces are present on the lateral and posterior surfaces of the sternum to articulate with the sternal ribs.

### 3.8 Pelvic girdle

The pubis (Pu; Figure 17) is a slender, paired bone with a triangular shape, featuring a small ventrally oriented process at its lateroventral corner. It articulates dorsally with the ilium, dorsoposteriorly with the ischium, and medially with its counterpart. The obturator foramen (Of; Figure 17b) is located dorsally on the pubis, ventral to the articulation between the three bones. The ilium (I; Figure 17) is a straight, paired bone oriented dorsoposteriorly, giving it a diagonal appearance. It features a laterally projecting process on its anterior facet and a posterior curvature, both positioned dorsally to the articulation region where the ilium articulates ventrally with the pubis and ischium.

**FIGURE 17:**
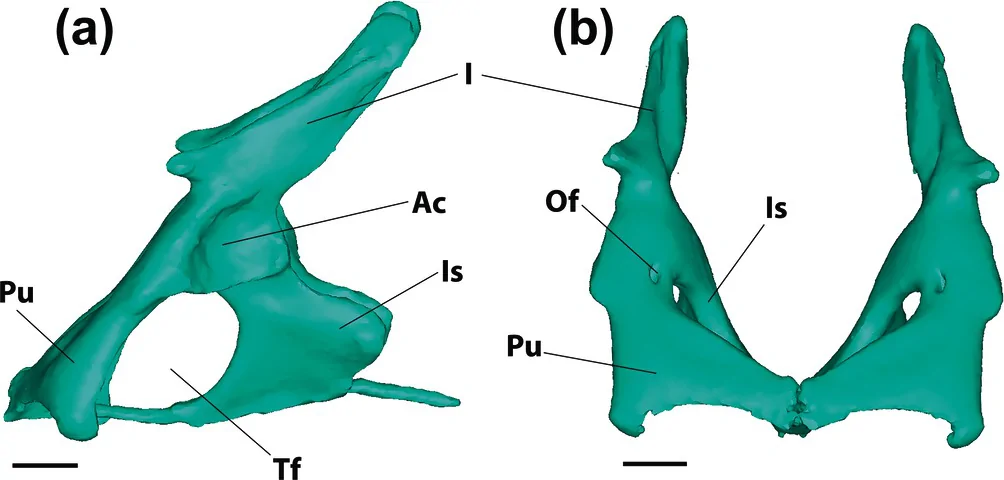
Pelvic girdle of the Ig04 specimen in left lateral view (a) and anterior view (b). Ac, Acetabulum; I, Ilium; Is, Ischium; Of, Obturator foramen; Pu, Pubis; Tf, Thyroid fenestra. Scale bars 1 cm.

The ischium (Is; Figure 17) is a paired bone with an irregular shape, characterized by an anterior concave curve and a posterior convex curve. On its ventral edge of the anterior facet, a thin, narrow process extends anteriorly, articulating with the medial articulation of both pubis bones through a cartilage structure. A mirrored process extends posteriorly. The thyroid fenestra is formed between the pubis and the ischium. At the region where all three pelvic bones meet, the acetabulum is located laterally, serving as the articular surface for the femur. Each bone contributes approximately one‐third to its overall structure.

### 3.9 Forelimb

The humerus (H; Figure 18a, b) is an elongated bone with lateral projections at its edges, forming a flattened region that is twice the width of the rest of the bone. At its distal edge, these projections create two rounded articular surfaces that articulate distally with the ulna and radius. Proximally, the humerus articulates with the procoracoid and measures approximately one‐third of the total forelimb length. The ulna and radius (U, Ra; Figure 18a, b) are slender bones, each measuring around one‐third of the forelimb length. Both exhibit slight lateral expansions at their edges, however less pronounced than those of the humerus. The ulna is slightly wider than the radius, featuring a broader proximal edge where it articulates with the humerus. The ulna is positioned medially and the radius laterally. Distally, the ulna articulates with the ulnare and pisiform bones of the carpals, while the radius articulates with the radiale.

**FIGURE 18:**
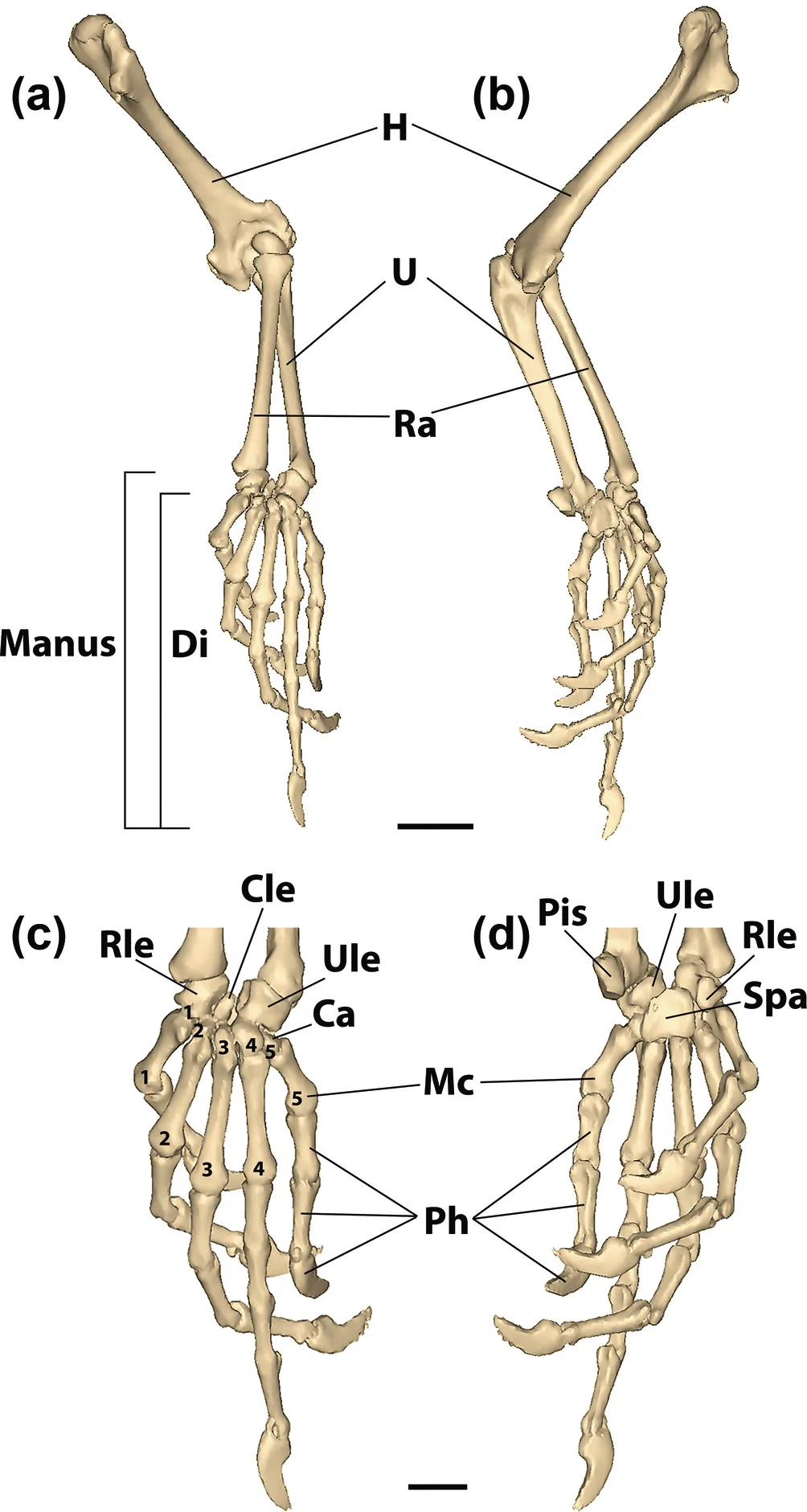
Forelimb of the Ig04 specimen in anterior view (a, c) and posterior view (b, d). Ca, Carpal; Cle, Centrale; Di, Digits; H, Humerus; Mc, Metacarpal; Ph, Phalange; Pis, Pisiform; Ra, Radius; Rle, Radiale; Spa, Palmar sesamoid; U, Ulna; Ule, Ulnare. Scale bars, 2 cm in (a, b), and 1 cm in (c, d).

The carpus consists of the ulnare, centrale, radiale, pisiform and five additional distal carpals. The ulnare (Ule; Figure 18c, d) is a rectangular bone featuring a concave process for proximal articulation with the ulna, as well as an additional concave process at its medial margin. Distally, it articulates with a carpal and medially with the centrale. The ulnare measures approximately one‐tenth of the length of the ulna. The pisiform (Pis; Figure 18c, d) articulates medially with the ulna, positioned proximally to the manus. Its shape is rectangular and measures three‐fourth the width of the ulnare. The centrale (Cle; Figure 18c, d) is an oval‐shaped bone located between the ulnare and radiale, articulating laterally with both and distally with a carpal. It measures approximately one‐third the width of the ulnare. The radiale (Rle; Figure 18c, d) is similar in shape and size to the ulnare, featuring a concave process for proximal articulation with the radius. It articulates medially with the centrale and distally with a carpal. The palmar sesamoid (Spa; Figure 18) exhibits a trapezoidal shape with a convex proximal margin and a concave distal margin. It presents the same width as the radiale, but twice its height. The palmar sesamoid is located at the ventral facet of the manus within the carpus; however, it does not articulate with any bone, as it is a sesamoid element, thus being evolved by the tendon of the flexor digitorum longus muscle.

There are five additional carpals (Ca; Figure 18c, d), which articulate proximally with the radiale, centrale and ulnare. Distally, each carpal articulates with a corresponding metacarpal. All the carpals exhibit similar oval shapes, measuring from approximately one‐fourth to two‐thirds of the size of the radiale. The digits (Di; Figure 18c, d) comprise five metacarpals (Mc; Figure 18c, d) and 17 phalanges (Ph; Figure 18c, d). Each metacarpal articulates with the most proximal phalange of its series, following a pattern from most medial to the most lateral digit: two, three, four, five and three phalanges per metacarpal. All phalanges share a long, slender and rectangular shape, as do the metacarpals, except for the first metacarpal, which exhibits a rounded lateral process in its proximal portion. The phalanges are generally uniform in length, measuring about one‐third of the longest metacarpal, and feature a ventral rounded process at their proximal end. The metacarpals range from one‐fifth to one‐third of the total length of the digit portion of the forelimb.

### 3.10 Hindlimb

The femur (Fe; Figure 19a, b) is a slender, elongated bone that exhibits a slight curvature along its length and expands laterally at its edges. It constitutes approximately one‐third of the total hindlimb length. Proximally, the femur articulates with the bones of the pelvic girdle via a condyle‐like, rounded edge measuring half the width of the bone. Adjacent to this articulation, a second rounded process is present, measuring around one‐third of the width of the bone. Distally, the femur articulates with the fibula through its expanded distal edge and laterally with the tibia.

**FIGURE 19:**
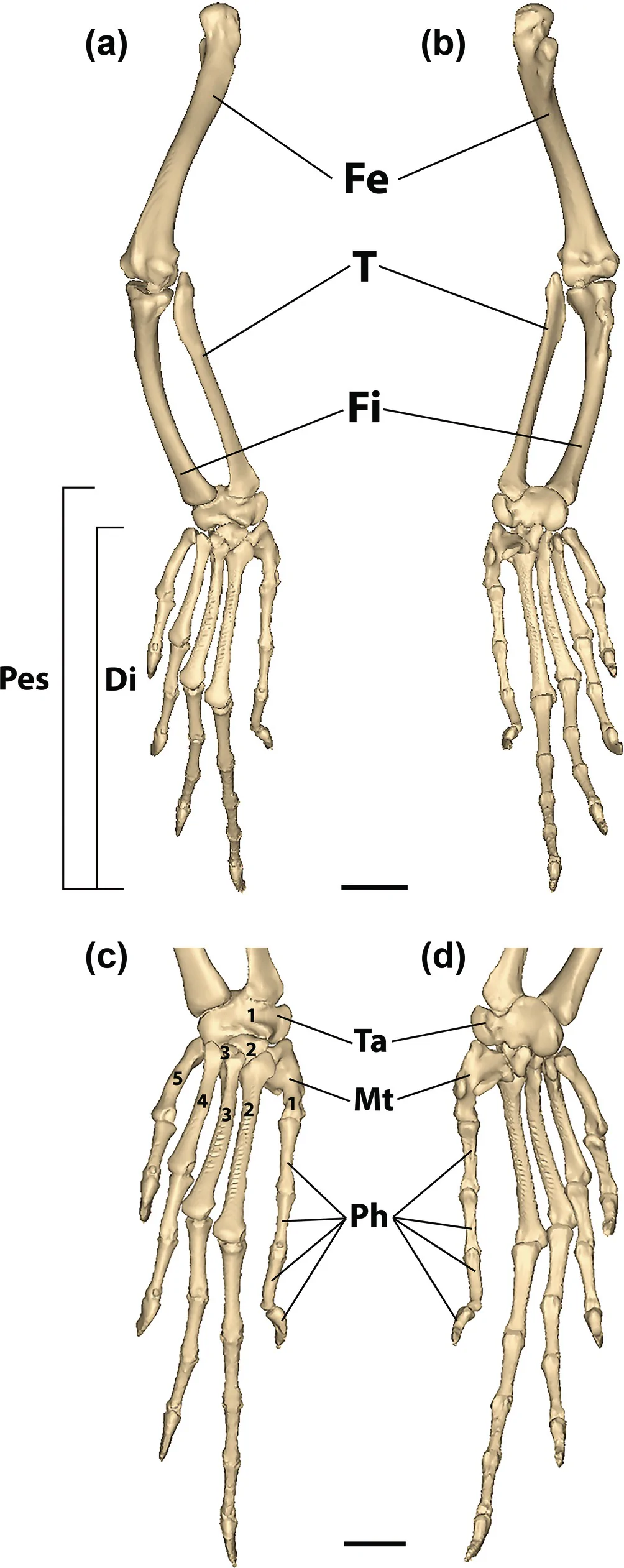
Hindlimb of the Ig04 specimen in anterior view (a, c) and posterior view (b, d). Di, Digits; Fe, Femur; Fi, Fibula; Mt., Metatarsal; Ph, Phalange; T, Tibia; Ta, Tarsal. Scale bars, 2 cm in (a, b), and 1.5 cm in (c, d).

The fibula (Fi; Figure 19a, b) is a slender bone similar to the femur, exhibiting a slight curvature but with less lateral expansion at its edges. It articulates proximally with the femur via a flattened plateau and distally with the tarsus. The fibula measures approximately one‐third of the total hindlimb length. The tibia (T; Figure 19a, b) is also a slender bone, equal in length to the fibula, but about half its width. Proximally, it articulates medially with the femur, while distally, it articulates with the tarsus. The fibula is positioned medially and the tibia laterally.

The tarsus consists of three tarsals. The first tarsal (Ta1; Figure 19c, d) is the most proximal and the largest, measuring the same width as the tarsus itself. It articulates proximally with the fibula and tibia, and distally with the tarsal 2 and 3, as well as with the metatarsal 1, 4 and 5. The first tarsal exhibits a rectangular shape and presents five processes: two concave processes at the proximal margin for articulation with the fibula and tibia, two concave processes at the distal margin for articulation with the other tarsals, and a convex projection at the lateral margin. The second tarsal (Ta2; Figure 19a–d) comprises a triangular shape and measures approximately one‐third the size of the first tarsal. It articulates distally with the tarsal 1 and laterodistally with the tarsal 3, as well as with the metatarsal 1, 2 and 3. The third tarsal (Ta3; Figure 19a–d) possesses an oval shape and measures one‐fourth the size of the tarsal 2. It articulates distally with the tarsal 1, laterally with the tarsal 2 and distally with the metatarsal 3 and 4.

The digits consist of the metatarsals (Mt; Figure 19a–d) and phalanges. While the number of metatarsals remains unchanged, the number of phalanges decreases slightly compared to the forelimb to 18. From the most medial to the most lateral digit, the phalangeal pattern is: two, three, four, five, and four phalanges per metatarsal. These bones exhibit the same shapes as those in the forelimb, with the process located in the proximal portion of the first metatarsal becoming larger, more prominent and laterally oriented. The metatarsals range from two‐fifth to one‐fifth of the total digit length.

### 3.11 Identification of ontogenetic stages

Before assessing individual variation, it is essential to establish the ontogenetic stages of each specimen. First, the number of dental rows in the pterygoid bones has been considered a putative indicator of maturity (Bochaton et al., 2016; de Queiroz, 1987). Among specimens, DZ UERJ, UF:Herp:181922 (Figures 1c, d and S1e, f), and OUVC 10677 exhibited a single dental row, indicative of juvenile traits. The MNRJ 12124 specimen was the only one to present two dental rows, an adult characteristic; specimens Ig01 and Ig04 could not be evaluated due to limitations in tomography. The orientation of the coronoid bone is also informative for ontogenetic assessment (Bochaton et al., 2016). Specimens Ig01 and Ig04 displayed a dorsal orientation, reflecting an adult stage, whereas DZ UERJ and UF:Herp:181922 (Figure 2a, b) exhibited a posterior orientation of the coronoid, consistent with juvenile traits. The OUVC 10677 presented a dorsal but slightly posterior orientation, though variation in this bone is common (Bochaton et al., 2016). The MNRJ 12124 specimen lacks the jaw and could not be assessed for this trait. The angular bone also undergoes ontogenetic changes, being positioned posterior to the teeth in juveniles and anterior in adults (Smith, 2009). Unlike other traits, the juvenile condition was not observed among the specimens. Both DZ UERJ and OUVC 10677 exhibited the angular anterior to the teeth, while the angular could not be evaluated in Ig01, Ig04, and UF:Herp:181922 due to tomography limitations.

Another ontogenetic change occurs in the shape of the parietal (Bochaton et al., 2016; de Queiroz, 1987), where specimens DZ UERJ and UF:Herp:181922 exhibited a larger medial constriction resembling a trapezoid (Figures 1a, b and S1a, b, and S7a, b), characteristic of juveniles; whereas Ig01, Ig04, MNRJ 12124 (Figure S7c, d), and OUVC 10677 displayed narrower medial constrictions, forming the Y‐shape typical of adults. Nevertheless, even among specimens with adult traits, variation was observed: Ig01 and Ig04 exhibited the narrowest parietal, with posterior processes presenting a more circular shape. The articulation between the jugal and squamosal also changes with maturity, being separated by the postorbital in lateral view in juveniles and eventually articulating in adults (Smith, 2009). Both DZ UERJ and OUVC 10677 exhibited the bones separated by the postorbital, whereas UF:Herp:181922 and MNRJ 12124 showed articulation ventral to the postorbital (Figures 1e, f and S1a–d). In MNRJ 12124, the contact is more pronounced, with the jugal dorsally overlapping the anterior process of the squamosal. Tomography limitations in Ig01 and Ig04 prevented accurate observation of this articulation.

According to Bochaton et al. (2016), the frontal bone changes shape throughout ontogeny. However, the interorbital constriction of the frontal is a subtle trait, making the delimitation of intermediary ontogenetic stages unclear; therefore, it was not considered in the present study. Both Smith (2009) and de Queiroz (1987) also report ontogenetic changes in the quadrate bone. Nonetheless, this trait was not included here, since its description in the available literature is not sufficiently detailed or illustrated to allow for consistent evaluation. Additionally, a nuchal fossa, which develops along ontogeny in the posterior region of the parietal bone, as reported by Paparella (2021), was not observed in the specimens.

Ontogenetic variations are also observed in the postcranial skeleton. According to Bochaton et al. (2016), the atlas elements in the *Iguana* genus fuse themselves. Among the specimens examined, only UF:Herp:181922 exhibited an unfused atlas (Figure S3g–i). Paparella (2021) reported that a similar ontogenetic fusion occurs in the axis of *I. iguana*; however, this feature was not observed in any of the specimens analyzed. Additionally, the long bones have their diaphysis and epiphysis fused (Bochaton, 2016). Both DZ UERJ and UF:Herp:181922 retained unfused long bones (Figure S8a, b), whereas Ig01, Ig03, Ig04, and Ig05 exhibited complete fusion. The fusion of the pelvic girdle bones also occurs during maturity (Bochaton, 2016); however, among specimens, it was observed only in Ig01 and Ig04 (Figure 17a, b). Certain ontogenetic changes reported by Bochaton et al. (2016) were not observed in any of the specimens, including the fusion of the odontoid process of the axis with the atlas centrum and the fusion of the sacral vertebrae. Additionally, Bochaton et al. (2016) does not report any ontogenetic variation in the caudal vertebrae.

Finally, the ontogenetic stages of the specimens could be determined to some extent. DZ UERJ and UF:Herp:181922, were identified as juveniles, as they exhibited predominantly juvenile traits, although they are not identical. These specimens differ in the fusion of the atlas (fused in DZ UERJ) and the articulation between the jugal and squamosal (present in UF:Herp:181922). Specimen OUVC 10677 displayed a mosaic of traits from both juvenile and adult stages, suggesting it may represent a more developed juvenile. Specimens MNRJ 12124, Ig01, Ig03, Ig04, and Ig05 were classified as adults based on the previously described features. The ontogenetic stage of Ig02 could not be determined, as the specimen consists solely of the pectoral girdle.

## 4 DISCUSSION

The literature on anatomical description, as well as intraspecific and ontogenetic variation, is well documented for iguanid (e.g., Conrad & Norell, 2007; Etheridge & de Queiroz, 1988; Oelrich, 1956; Smith, 2009) and iguanine (e.g., de Queiroz, 1987; Lai & Scarpetta, 2024; Paparella & Caldwell, 2021) lizards. Several studies have documented considerable variation within lizard species across various families, including Chamaeleoninae (e.g., Rieppel & Crumly, 1997) and Lacertidae (e.g., Barahona et al., 1998; Tschopp, 2016), as well as more broadly within Lepidosauria (e.g., Evans, 2008). Moreover, morphological and ontogenetic differences within the *Iguana* genus have also been studied (Bochaton et al., 2016; Conrad & Norell, 2010). Building on this framework, we report novel cases of intraspecific morphological variability in *I. iguana* specimens particularly regarding skull bones, including the composition of the parietal foramen, the presence of a neural spine in the atlas, the number of cervical ribs, dorsal vertebral and rib counts, and the shape of several skeletal structures. Particularly, the caudal region exhibited the greatest morphological variation, involving the absence of double transverse processes in one specimen, previously described as characteristic of *I. iguana* (Etheridge, 1967), the specific vertebra where the haemal arch originates, and the occurrence of a cone‐like structure in the last caudal vertebra.

### 4.1 Identification of ontogenetic stages

According to Evans (2008), intraspecific variation in lizards may result from subtle differences in skeletal development, which can lead, for example, to two similarly sized skulls exhibiting bone elements at different developmental stages or to the presence or absence of key traits within species. Consequently, all variation found in the skeletal structures among multiple adult individuals is considered intraspecific variation in the following sections.

These findings indicate that previously used traits for estimating ontogenetic stage, such as pterygoid dentition and bone fusion, remain reliable but may vary among individuals due to delayed or accelerated ossification. This variation between stages and specimens highlights the challenge of accurately assessing intermediate ontogenetic stages and, consequently, the need for updated and detailed descriptions of these stages in *I. iguana*. Furthermore, some of these features are shared with other taxa, for instance, the adult Y‐shaped parietal condition in *I. iguana* is also found in the iguanines *Conolophus*, *Cyclura*, and *Amblyrhynchus* (Etheridge & de Queiroz, 1988; Paparella, 2021). In addition, the dorsal overlapping between the jugal and squamosal in specimen MNRJ 12124 suggests that some adult characteristics of *I. iguana* may converge with those described as occurring only in the acrodontan iguanians *Pogona* and *Physignathus* (Paparella, 2021).

Therefore, the morphological diversity observed among adult specimens of *I. iguana* likely reflects intraspecific variability rather than taxonomic distinction. This emphasizes the importance of considering ontogenetic context in morphological studies, especially when evaluating skeletal traits that change throughout development.

### 4.2 Skull bones

The shape and relative size of the nasal and frontal bones appears to be associated and varied among specimens. In specimens MNRJ 12124 and OUVC 10677, the nasal exhibits a rectangular shape, whereas in the DZ UERJ and UF:Herp:181922 specimens, it is triangular. In the MNRJ 12124 specimen, the nasal matches the length of the frontal and measures half its width, with the frontal occupying one‐fourth of the skull roof length (Figure S7). In contrast, the DZ UERJ specimen presents a nasal approximately one‐third the length of the frontal, which spans about half the skull roof length. In the OUVC 10677 and UF:Herp:181922 specimens, the nasal is two‐thirds the length of the frontal, which constitutes about one‐third of the skull roof length. Thus, smaller nasals were associated with larger frontals (Figure S7a, b), whereas proportionally larger nasals occurred with frontals of similar size (Figure S7c, d). Considering the ontogenetic stage of the specimens, these results suggest that juveniles may exhibit a small triangular‐shaped nasal (Figure S7a, b). OUVC 10677, considered a late juvenile, displays an intermediate condition. Additionally, the nasal bone exhibits plate‐like ornamentation, which varies its intensity among specimens, with the MNRJ 12124 specimen showing the most pronounced and detailed ornamentation (Figure S7c). This feature appears to be more developed in adult individuals.

The high degree of shape and size variation observed in the frontal region here is also noted by Conrad and Norell (2010). However, while those authors suggest that a wider frontal is associated with larger specimens, our findings reveal an opposite pattern. The DZ UERJ specimen, with a skull length of 4.8 cm, displays a proportionally larger frontal bone (Figure S7a, b), while the MNRJ 12124 specimen, measuring 8 cm, has a relatively smaller frontal (Figure S7c, d). The UF:Herp:181922 specimen, with a skull length of 5.8 cm, displays a frontal bone size that is intermediate between these two extremes (Figure 1a, b). This suggests an inverse association in our specimens, where larger specimens exhibit a proportionally smaller frontal bone. Size differences may also reflect ontogenetic stage, as juveniles display larger frontals and adults smaller ones.

The parietal foramen differs in its contribution by the frontal and parietal bones. In the DZ UERJ and UF:Herp:181922 specimens, the foramen is equally divided between the two bones (Figures 1a, b and S7a, b). In the specimen MNRJ 12124, the frontal contributes two‐thirds of the foramen's structure (Figure S7c, d). However, in the OUVC 10677 specimen, the parietal foramen is formed entirely by the parietal bone. This variation in the parietal foramen is consistent with de Queiroz (1987), as well as Conrad and Norell (2010) and Paparella (2021), who reported that, although most individuals exhibit the foramen at the frontoparietal suture, it is common for it to lie predominantly within either the parietal or frontal bone. However, de Queiroz (1987) refers to cases in other species where the foramen is formed entirely by one of these bones, and does not specifically address the *Iguana* genus.

Variation in other skull bones was also observed among specimens. The prefrontal, for instance, ranged from half the size of the frontal (DZ UERJ) to two‐thirds (MNRJ 12124 and UF:Herp:181922; Figures 1 and S7) and four‐fifth (OUVC 10677). The presence of two bosses connected by a crest in the prefrontal of the *I. iguana*, as reported by Paparella (2021), was not observed in the specimens examined here. The postfrontal showed variability in size and shape, even within the same individual, ranging from the same size (DZ UERJ) or one‐third (OUVC 10677) of the premaxilla to one‐fourth of the prefrontal (MNRJ 12124; Figure S7). Since these features vary among juveniles as well as among adults, variation in size of the prefrontal and postfrontal bones may represent intraspecific variation. The maxilla exhibited variability in tooth count, with DZ UERJ presenting 18 teeth, UF:Herp:181922 and OUVC 10677, 22 teeth, and MNRJ 12124, 23 teeth. The palatine length ranged from two‐sevenths of the skull (MNRJ 12124) to one‐fourth (DZ UERJ and OUVC 10677), suggesting a possible reduction during ontogenetic development. Additionally, the circular anterior process observed in the septomaxilla of the UF:Herp:181922 specimen (Figure S1e, f) has not been previously reported in the literature and could not be observed in other specimens (OUVC 10677 and DZ UERJ) due to their tomography resolution. The remaining skull bones are consistent across specimens. The skull of Ig01 and Ig04 could not be analyzed due to the tomography resolution.

### 4.3 Vertebral column

The atlas showed significant variation across specimens. In the Ig04, UF:Herp:181922 and OUVC 10677 specimens, the neural spine is absent (Figure S3a–c, g–i), an observation not previously documented in the literature for *I. iguana* (Arantes, 2016; Lima, 2015). In *Amblyrhynchus*, the atlas presents a neural spine (Paparella, 2021). However, the absence of a neural spine has been documented in other lizard species (Barahona et al., 1998; Čerňanský, 2016; Čerňanský et al., 2019; Paparella, 2021; Silva & Verrastro, 2007). Conversely, the Ig01, Ig05 and DZ UERJ specimens displayed a small, triangular neural spine in the atlas vertebra, which exhibits a steep anterior surface and a postero‐dorsally inclined surface, measuring approximately 1/12 of the vertebra's height (Figure S3d–f). Additionally, the UF:Herp:181922 specimen uniquely exhibited a fissure in the ventral one‐fourth of its vertebral body height (Figure S3g–i), a feature associated with the unfused atlas in juvenile iguanas (Bochaton, 2016) and in iguanine cycluras (Paparella, 2021). However, this feature is found in the extant apodal squamate group Dibamidae (Čerňanský & Stanley, 2019) as well as the anguid genera *Pseudopus*, *Ophisaurus,* and *Anguis* (Čerňanský et al., 2019). A transverse ligament within atlas's vertebral foramen was not observed here, probably due to their tomography resolution, although it is reported for *I. iguana* in previous studies (Arantes, 2016; Lima, 2015; Silva & Verrastro, 2007; Veronese & Krause, 1997), as well as in other iguanines (Oelrich, 1956). Variability was also noted in the anterior end of the neural spine in the axis, since it extends up to the anterior limit of the odontoid process in the Ig04 specimen, and reaches only the anterior limit of the cotyle in Ig01, UF:Herp:181922 and OUVC 10677 specimens.

In the cervical vertebrae the length of the vertebral centrum decreases progressively, starting more elongated in C3 and C4, similar to the axis, and gradually decreases until C8, where it is about one‐seventh shorter than the axis in the UF:Herp:181922, Ig01, Ig04, and Ig05 specimens. In the DZ UERJ specimen, however, the cervical vertebrae are relatively uniform in length, measuring around 8 mm each, which may represent a juvenile stage. The dorsal cume of the neural spine in C3 to C8 varies intraspecifically among specimens, ranging from sharp angles (Ig01 and UF:Herp:181922) to rounded edges (Ig04, DZ UERJ and OUVC 1677). The presence of eight cervical vertebrae is consistent in lacertids and iguanine lizards, whereas chameleons exhibit five (de Queiroz, 1987; Smith, 2009; Tschopp, 2016).

The number of cervical ribs varied among specimens: the DZ UERJ, Ig01, Ig04 and Ig05 specimens exhibited in four cervical vertebrae (from C5 to C8), whereas the UF:Herp:181922 specimen, presented in five vertebrae (from C4 to C8). This is consistent with Etheridge and de Queiroz (1988), who reported that iguanids exhibit cervical ribs with the third, fourth, or fifth vertebra. The morphology of the ribs in C4 is characterized by its short length, measuring approximately two‐thirds of the vertebral width, and a slight posterior curvature. The shape and size of the remaining cervical ribs remained similar across specimens, however, the rib in C6 exhibited variation in its distal end, which could be either flattened with a curved rectangular shape (Ig01, UF:Herp:181922, and DZ UERJ) or a circular shape (Ig04). Additionally, a cartilaginous structure at the distal end of the ribs from C5 to C8 was observed in the UF:Herp:181922 specimen, as well as in DZ UERJ, except for the C8. This structure was also found in the C6 of Ig01 and Ig04; however, due to tomography limitations, it is not possible to determine its presence in other vertebrae. This cartilaginous structure occurs in iguanids as reported by Etheridge and de Queiroz (1988).

Among the dorsal vertebrae, the cotyle exhibited variation across individuals. The D1 vertebra presented a consistent cotyle across specimens. In Ig04, the cotyle of D5 was over four times the size of the vertebral foramen, however in Ig01, it presented two times that size. For the D12 vertebra, the cotyle was more than six times the size of the vertebral foramen in Ig04, while in Ig01, it was three times larger. Paparella (2021) reported that iguanine *Ctenosaura* and *Iguana* exhibit a ventral crest between the pleurapophysis and the cotyle in the cervical vertebrae bearing ribs and in the first dorsal vertebrae. In *I. iguana*, however, this crest was described as ending after the second dorsal vertebra, in contrast to its occurrence up to D12 in the specimen analyzed here, which resembles the condition described for *Ctenosaura*. Paparella (2021) also mentioned the occurrence of a similar feature involving the condyle, but it was not observed in any specimens examined here. In the Ig01 specimen, the condyle was half larger than the vertebral foramen in D1 and approximately three times larger in D12. While in the Ig04 specimen, the condyle was twice the size of the vertebral foramen in both D1 and D5. Regarding the postzygapophysis, it appeared smooth and rounded in Ig04, while more pointed with lateral spikes in Ig01.

The number of ribs reported in the literature also varies. Lima (2015) describes seven sternal ribs for *I. iguana*, while Brainerd et al. (2016) report only four sternal ribs. The discrepancy with Brainerd et al. (2016) is due to their consideration of only the direct sternal ribs as sternal ribs. Nonetheless, following Lima (2015), we also considered the ribs indirectly connected to the sternum as sternal ribs, resulting in a total of six sternal ribs. Our consideration relies on the fact that these indirect ribs also articulate with the sternum and share the same morphology as the direct sternal ribs, exhibiting a cartilaginous sternal segment, thus being considered as sternal ribs. The four direct sternal ribs noted in both Lima (2015) and Brainerd et al. (2016) are consistent with the observations in the Ig01, Ig02, Ig03, Ig04, Ig05 and DZ UERJ specimens, except for UF:Herp:181922, where only three direct sternal ribs were found. Although this condition was observed in a juvenile specimen, we interpret it as intraspecific variation, since no additional vertebrae are formed during ontogeny. Furthermore, squamates possess four direct sternal ribs attached to vertebrae nine through 12, a condition consistent with our observations (Etheridge & de Queiroz, 1988; Smith, 2009).

There is variation in the number of ribs present in the rib cage among specimens. In the DZ UERJ, Ig01, Ig03, Ig04, and Ig05 specimens, four vertebrae have direct sternal ribs and two have indirect sternal ribs, totaling six sternal rib vertebrae. In contrast, the UF:Herp:181922 specimen exhibits three vertebrae with direct sternal ribs and two with indirect sternal ribs, resulting in a total of five sternal rib vertebrae. The Ig01, Ig04, DZ UERJ, and UF:Herp:181922 specimens exhibit two vertebrae with long free vertebral ribs. However, due to tomography resolution limitations, only one vertebra with a free rib could be observed in specimens Ig02 and Ig03. In the Ig05 specimen, there is also only one long free vertebral rib, which is followed by a series of short vertebral ribs until the sacral series, a pattern also observed in the other specimens except for Ig02 and Ig03. In the UF:Herp:181922 specimen, the D7 and D8 nearly connect distally at their cartilaginous extremities, almost forming a “V” shape. However, in Ig01, Ig02, Ig03, Ig04, and DZ UERJ, the distal ends of D7 articulate with each other, while in D8 they do not, this observation is consistent with Lima (2015). The remaining eight free rib vertebrae are similar across the Ig01, Ig04, Ig05, DZ UERJ, and UF:Herp:181922 specimens. The total number of dorsal vertebrae is 16 vertebrae in the Ig01, Ig04, and DZ UERJ specimens and 15 vertebrae in the UF:Herp:181922 and Ig05 specimens.

The presence of two sacral vertebrae is a consistent feature in iguanines (de Queiroz, 1987). Within the specimens analyzed herein, the shape of the vertebral foramen of Sa1 varied from a circular shape (Ig04) to a triangular shape (Ig01 and UF:Herp:181922). In contrast, the vertebral foramen of S2 remained oval‐shaped across the Ig01, Ig04, and UF:Herp:181922 specimens. The length of the vertebral centrum of the sacral vertebrae also showed variation; the UF:Herp:181922, Ig04 and DZ UERJ specimen exhibited a centrum approximately three‐fourths the length of the last dorsal vertebra's centrum, while in the Ig01 specimen, it was one‐half of that length.

Findings regarding the high variability of caudal vertebrae in *I. iguana* are not reported in the literature. While variability in shape and order among lizard families and genera is discussed, with Iguaninae exhibiting considerable variation in caudal vertebrae (de Queiroz, 1987), intraspecific variability in *I. iguana* remains less debated (Etheridge, 1967). Notwithstanding, while significant, tails are still poorly studied (Schwaner et al., 2021). Most research on lizard tail anatomy focuses on tail autotomy (e.g., Barr et al., 2019, 2022; Gordeev et al., 2020; Ritzman et al., 2012), tail bifurcation (e.g., Arrivillaga & Brown, 2020; Baum & Kaiser, 2024), or taxa with reduced limbs (e.g., Holovacs et al., 2020). Moreover, no studies were found discussing ontogenetic variation in the caudal vertebrae of *Iguana*. In the lizard *Varanus komodoensis*, tail length is proportionally larger relative to snout‐vent length in juveniles than in adults; however, because not all specimens in the present study exhibited complete tails, this could not be assessed (Tomańska et al., 2024). Ontogenetic variation in caudal vertebrae has been described in relation to autotomy in some lygosomine species (Barr et al., 2022) and in vertebral laminae in lacertid species (Tschopp, 2016). Therefore, the variations observed herein were considered as possible ontogenic variation.

According to Etheridge (1967), within the Iguanidae family, there are four types of caudal sequence, with the one present in *I. iguana* displaying four morphotypes that differ based on the presence and number of transverse processes. The Ig04 and UF:Herp:181922 specimens exhibit the morphotypes as described by Etheridge (1967, fig. S9), however, they display differences when analyzed according to the classification employed in the present study. This contrast arises from the inclusion of the neural spine and haemal arch in our analysis, which were not considered in Etheridge's work. Both specimens exhibit differences in the presence of morphotype 1 and 2, as well as 6 and 7, which are distinguished by the presence of the haemal arch, but considered as the same morphotype by Etheridge (1967). Additionally, in the Ig04 specimen, two distinct morphotype orders without a transverse process were observed: one with the presence of neural spine and haemal arch (morphotype 5) and another without either structures (morphotype 7). Furthermore, the presence of the haemal arch varied among specimens; the UF:Herp:181922 specimen exhibited it from the first caudal vertebra, while in the Ig01 and Ig04 specimens, it appeared only from the second and third vertebra, respectively. The presence of a haemal arch on the first caudal vertebra of UF:Herp:181922 contrasts with de Queiroz (1987), who reported that, in iguanine lizards, the first haemal arch is positioned on the second or third caudal vertebra, as observed in the other specimens, Ig01 and Ig04, both in the second vertebra. In *V. komodoensis*, for example, the first haemal arch appears only on the second caudal vertebra (Tomańska et al., 2024). The absence of a haemal arch on the first vertebra is associated with increased caudal mobility, which is pivotal for locomotion and behavior (Tomańska et al., 2024), and this variation may therefore influence the habits of the UF:Herp:181922 specimen.

The pattern found in the Ig01 specimen also contrasts with that described by Etheridge (1967). In this specimen, the morphotype 3, characterized by the presence of two transverse processes, was absent. However, Etheridge (1967) asserts that the presence of these processes is characteristic of *I. iguana*. Since this variation was observed in an adult specimen, we interpret the presence of two transverse processes in *I. iguana* as susceptible to intraspecific variation. Regarding the diagnosis of this species, all specimens are consistent with Conrad and Norell (2010), as they all exhibit a single transverse process on the caudal vertebrae and a pseudospine crest.

The most significant intraspecific morphological variation in *I. iguana* was observed in the caudal structure, as all specimens exhibited distinct patterns in the caudal structure, specifically regarding the morphotypes present in each caudal region. The Ig01 specimen displayed only three morphotypes (also due to its incomplete tail): 1, 2 and 5. The Ig04 specimen presented nearly all morphotypes, comprising six: 1, 2, 3, 4, 5 and 7. Lastly, the UF:Herp:181922 specimen exhibited five morphotypes: 2, 3, 4, 5 and 6. Notably, the UF:Herp:181922 was the only specimen with a complete tail structure and contained 58 caudal vertebrae; similar to Lima (2015), that identified from 60 to 70 caudal vertebrae in adults of *I. iguana*. However, Filek et al. (2025) reported only 39 caudal vertebrae in *I. iguana*, whereas de Queiroz (1987) observed 70. Nevertheless, the latter two do not provide information on ontogenetic stage. Consequently, the number of caudal vertebrae can be considered in having some degree of variation.

The morphotype 1 was observed in the first caudal vertebra of the Ig01 specimen and in the first two caudal vertebrae of the Ig04 specimen. Its zygosphene shape varied from rounded (Ig04 specimen) or straight (Ig01 specimen). The morphotype 2 extends across a varying number of vertebrae depending on the individual specimen. In the Ig01 specimen, it begins at Ca2 and continues until Ca14. In the Ig04 specimen, it spans from Ca3 to Ca10. The UF:Herp:181922 specimen differs from the others, with morphotype 2 present from the first caudal vertebra to Ca10. In its first vertebrae, it displays a wider vertebral centrum similar to morphotype 1. In *V. komodoensis*, the first caudal vertebrae resemble the first vertebra observed in the *I. iguana* specimens, exhibiting a wide centrum and neural arch, as well as horizontal transverse processes (Tomańska et al., 2024). Along the caudal segment, the centrum decreases in width in both species, however, *V. komodoensis* displays vertical transverse processes in the transversal part of its tail, contrasting with the horizontal orientation observed in *I. iguana* (Tomańska et al., 2024). Although being a squamate of reduced limbs, the gymnophthalmid lizard *Calyptommatus leiolepis* exhibits neural spines similar to those of *I. iguana*, with both species showing an increase in spine length in caudal vertebrae relative to sacral and presacral vertebrae (Holovacs et al., 2020). The neural spines of the saurian lizard *Anolis carolinensis* also resemble those of *I. iguana*, as in both species the spines originate at the middle of the centrum and eventually shift towards the posterior portion of the vertebra (Ritzman et al., 2012).

The pseudospine crest, featured in the morphotype 2, was observed in seven vertebrae in the Ig01 specimen, five vertebrae in the Ig04 specimen and six vertebrae in the UF:Herp:181922 specimen. These findings are consistent with de Queiroz (1987), who reported a variable pseudospine crest in iguanine lizards that does not extend beyond the tenth caudal vertebra. This crest may vary in size among individuals or along the caudal extension but never exceeds one‐fourth of the vertebral centrum's height.

The morphotype 3 occurs within a limited series of vertebrae: in the Ig04 specimen, it spans from Ca11 to Ca13, while in the UF:Herp:181922 specimen, it is observed from Ca11 to Ca12. In the Ig01 specimen, small lateral protuberances were identified on the vertebral centrum of Ca13, corresponding to the location where the anterior process would typically be present; however, these were not classified as a morphotype 3 due to their underdeveloped nature. The presence of two transverse processes in caudal vertebrae occurs in various Squamate groups, such as lacertids (Tschopp, 2016) and gymnophthalmids (Holovacs et al., 2020), but is absent in others, including varanids (Tomańska et al., 2024) and anoles (Ritzman et al., 2012). In *I. iguana*, the fracture plane for autotomy lies between the two transverse processes or posterior to the single anterior process of the morphotype 4 defined here (Etheridge, 1967); however, this feature was not observed in the specimens, probably due to tomography limitations. In lacertids, the fracture plane is similar to that in *I. iguana*, but extends to vertebrae lacking transverse processes, like morphotype 5 (Tschopp, 2016). In *A. carolinensis*, the fracture plane occurs only in vertebrae without transverse processes (Ritzman et al., 2012), whereas in *C. leiolepis* it is present only in the last caudal vertebra (Holovacs et al., 2020). In contrast, the scincid lizard *Egernia kingii* exhibits fracture planes in all caudal vertebrae (Barr et al., 2022).

It is not possible to determine with certainty how many vertebrae exhibit the morphotype 4 in the Ig04 specimens due to a rupture in its tomography. However, it is identified in Ca14 and Ca15, where the tail is divided. In the UF:Herp:181922 specimen, this morphotype is present in two vertebrae: Ca13 and Ca14. In this case, the transverse processes are much smaller than those in Ig04, manifesting only small lateral protuberances. This appears to be a transitional morphotype between types 3 and 5, found in only a few vertebrae. This morphotype, retaining only the anterior transverse process, is consistent with the observations of Etheridge (1967) and de Queiroz (1987). Another feature of this morphotype is the progressively more inclined neural spines, a trait also observed in *V. komodoensis* (Tomańska et al., 2024).

The morphotype 5 is present in all specimens: in the Ig01 specimen, it appears in four vertebrae from Ca15 to Ca19 (where the tails is incomplete); in the Ig04 specimen, it is found in 10 vertebrae (the total number of the vertebrae cannot be determined due to an anterior rupture of the tail); and in the UF:Herp:181922 specimen, it begins at Ca15 and extends through 27 vertebrae, ending at Ca42. The occurrence of vertebrae with two transverse processes (as in morphotype 3), followed by a single transverse process (morphotype 4) and vertebrae lacking transverse processes (morphotype 5), is also observed in lacertids (Tschopp, 2016).

The morphotype 6 was only identified in the UF:Herp:181922 specimen, and the cone‐like structure featured in its last vertebra, Ca58, was exclusive to this specimen. The morphotype 7 was also found in one specimen, the Ig04, and notably, its last vertebra does not exhibit the cone‐like structure observed in morphotype 6. Although this cone‐like structure was observed only in a juvenile specimen, we interpret it as intraspecific variation, since it has been previously reported in *I. iguana* (Filek et al., 2025).

### 4.4 Girdles

The presence of a scapular fenestra varies among lizard species, being observed in *Iguana*, the iguanian *Uranoscodon*, *Anolis*, and the iguanine *Amblyrhynchus* (Paparella, 2021). The arrow‐like shape of the interclavicle remains consistent across all specimens; however, its curvature varies, ranging from a nearly straight angle (Ig04) to an acute angle (Ig02, Ig03). The Ig01, UF:Herp:181922, and DZ UERJ specimens exhibit curvatures that fall between these extremes. In all specimens (UF:Herp:181922, Ig01, Ig02, Ig03, and Ig04), except for DZ UERJ, the most posterior point of the interclavicle is surrounded by a foramen, which varies in size from one‐fifth to three‐fourth of the width of the interclavicle. In the DZ UERJ specimen, a foramen is also present, but differs in shape, being circular and located posterior to the interclavicle, measuring approximately one‐fourth of its width. The lateroventral process found in the procoracoid is only present in Ig04, while it is absent in the DZ UERJ, Ig01, Ig02, Ig03, Ig05, and UF:Herp:181922 specimens. The sternum remains generally consistent among specimens, varying only in the UF:Herp:181922, where it exhibits only eight articular surfaces for the sternal ribs.

The variation observed in the pelvic girdle concerns the length of the ventral process of the ischium. In the Ig01, Ig04, and DZ UERJ specimens, the anterior portion of this process articulates with the pubis. However, in the UF:Herp:181922, the process is only half the length that in the other specimens and does not articulate with the pubis, which may be interpreted as an ontogenetic variation. This process is connected to the ischium by two cylindrical structures, one on each bone, which are also observed in the iguanines *Cyclura* and *Ctenosaura* (Paparella, 2021).

### 4.5 Limbs

The bones of both forelimbs and hindlimbs, including carpal and tarsal elements, displayed no significant variations across specimens (DZ UERJ, Ig01, Ig04, Ig05, and UF:Herp:181922), where these structures were present. Specimens Ig01 and Ig04 fully conformed to the previously described morphology, while UF:Herp:181922 and DZ UERJ presented unfused epiphysis in their long bones, indicating their juvenile status, as discussed in the section on ontogenetic stage identification (Figure S8).

## 5 CONCLUSIONS

Here, we provide a significant and updated anatomical revision of *I. iguana*, focusing on both its cranium and axial skeleton. Notably, a high degree of individual variability was observed in *I. iguana*, particularly in the skull, such as differences in bone proportions and in the composition of the parietal foramen, and in the vertebral column, including the presence of a neural spine in the atlas vertebra, variation in the number of cervical and dorsal ribs, and in dorsal vertebral count. The most significant morphological variation was observed in the caudal vertebrae, where we report variation in the occurrence of double transverse processes, previously regarded as pivotal for the species, the vertebral position of the haemal arch emergence, and the presence of a cone‐like structure in the last vertebra. Based on these findings, we define new caudal morphotypes for the species that enhance current knowledge of the skeletal anatomy of the species.

The data described here provide evidence of intraspecific variation in *I. iguana*. Many traits susceptible to intraspecific variation were observed in this study, highlighting the need for further research on variation in iguanas to enhance our knowledge of the group's anatomy. Therefore, a larger sample size, together with datasets of higher‐resolution CT scans, will be essential for conducting more robust comparative analyses in the future. Future research should also prioritize studies on the rates of development of different traits across ontogenetic stages, in order to better understand which characters change and the timing of these changes throughout the *I. iguana* development. This would allow for a clearer definition of ontogenetic stages of the species, since intermediate traits are often imprecise, which hinders the evaluation of developmental stages in some specimens.

## AUTHOR CONTRIBUTIONS

**Vieno Rosa:** Investigation; writing – original draft; methodology; validation; writing – review and editing; software; visualization; formal analysis. **Marcos Vinícius Coelho:** Conceptualization; investigation; writing – original draft; methodology; validation; visualization; writing – review and editing; software; formal analysis. **Camila Cupello:** Conceptualization; investigation; funding acquisition; writing – original draft; supervision; resources; software; data curation; writing – review and editing; visualization; validation; methodology; formal analysis; project administration.

## FUNDING INFORMATION

Vieno Rosa was supported by the Coordenação de Aperfeiçoamento de Pessoal de Nível Superior—Brasil (CAPES)—Finance Code 001 and grant number 88887.142430/2025‐00. Fundação Carlos Chagas Filho de Amparo à Pesquisa do Estado do Rio de Janeiro (FAPERJ) supported Vieno Rosa (grant No. SEI‐260003/006741/2022, E‐26/202.054/2022); Marcos Vinícius Coelho (grant No. SEI‐260003/009221/2022, E‐26/203.217/2022); and Camila Cupello (grants no. SEI‐260003/001594/2022, E‐26/210.369/2022 and SEI‐260003/002247/2022, E‐26/200.605/2022).

## CONFLICT OF INTEREST STATEMENT

The authors declare that no conflict of interest exists.

## Supporting information


**FIGURE S1.** Skull interior of the UF:Herp:181922 specimen: (a) drawing of ventral view of the interior of the skull, (b) ventral view of the interior of the skull, (c) drawing of left lateral view of the interior of the skull, (d) left lateral view of the interior of the skull, (e) drawing of left lateral view of the interior of the skull and medial view of the jaw with squamosal and supratemporal sectioned together, (f) left lateral view of the interior of the skull and medial view of the jaw with squamosal and supratemporal sectioned together, (g) drawing of posterior view of the interior of the skull, (h) posterior view of the interior of the skull. An, Angular; Art, Articular; Bo, Basioccipital; Co, Coronoid; D, Dentary; Ec, Ectopterygoid; Ep, Epiterygoid; Fr, Frontal; J, Jugal; L, Lacrimal; Mx, Maxilla; N, Nasal; Ot, Otooccipital; P, Parietal; Pa, Palatine; Pfr, Postfrontal; Po, Postorbital; Prf + L, Prefrontal and Lacrimal; Pro, Prootic; Pt, Pterygoid; Px, Premaxilla; Q, Quadrate; San, Surangular; Sep, Septomaxilla; So, Supraoccipital; Sp, Sphenoid; Sq, Squamosal; Spl, Splenial; St, Supratemporal; V, Vomer. Dashed lines, inferred sutures based on the literature and other specimens. Blue, cartilaginous structure. Scale bars, 0.4 cm.

**FIGURE S2.** Neurocranium of the UF:Herp:181922 specimen: (a) drawing of anterior view, (b) anterior view, (c) drawing of posterior view, (d) posterior view, (e) drawing of left lateral view, (f) left lateral view, (g) drawing of ventral view, (h) ventral view, (i) drawing of dorsal view, (j) dorsal view. Bo, Basioccipital; Ot, Otooccipital; Pro, Prootic; So, Supraoccipital; Sp, Sphenoid. Dashed lines, inferred sutures based on the literature and other specimens. Scale bars, 0.4 cm in (a, b, c, d) and 0.2 cm in (e, f, g, h, i, j).

**FIGURE S3.** Atlas vertebra: (a–c) Ig04, (d–f) Ig01 and (g–i) UF:Herp:181922 specimens in anterior, left lateral, and dorsal view. Hyp, Hypapophysis; Ns, Neural spine; Poz, Postzygapophysis; Prz, Prezygapophysis; Tpa, Anterior transverse process; Tpp, Posterior transverse process; Vf, Vertebral Foramen. Scale bars, 0.3 cm in (a, b), 0.2 cm in (c, g–i), 0.5 cm in (d–f ).

**FIGURE S4.** Cartilaginous structure in the distal region of the DZ UERJ specimen. The triangle indicates the structure. Scale bar 1 cm.

**FIGURE S5.** Dorsal vertebrae of the Ig04 specimen: (a–c) D1, (d–f) D5 and (g–i) D12 in anterior, left lateral and posterior view. Con, Condyle; Cot, Cotyle; Cp, Cartilaginous portion of the rib; Ns, Neural spine; Pij, Putative intracostal joint of rib; Ple, Pleurapophysis; Poz, Postzygapophysis; Prz, Prezygapophysis; R, Rib; Vf, Vertebral Foramen; Vp, Vertebral portion of the rib; Za, Zygantrum; Zs, Zygosphene. Scale bars, 0.7 cm in (a, b), 1 cm in (c), and 0.8 cm in (d–i).

**FIGURE S6.** Sacral vertebrae of the Ig04 specimen: (a–c) S1 and (d–f) S2 in anterior, left lateral and dorsal view; (g) S1 and S2 in left lateral view. Con, Condyle; Cot, Cotyle; Ns, Neural spine; Ple, Pleurapophysis; Poz, Postzygapophysis; Prz, Prezygapophysis; S1, Sacral 1; S2, Sacral 2; Vf, Vertebral Foramen; Za, Zygantrum; Zs, Zygosphene. Scale bars, 1 cm in (a, b, d, e), 0.9 cm in (c, f), and 0.6 cm in (g).

**FIGURE S7.** Nasal (red) and Frontal (purple) variation in DZ UERJ (a, b) and MNRJ 12124 (c, d) specimens. Ec, Ectopterygoid; Fr, Frontal; J, Jugal; L, Lacrimal; Mx, Maxilla; N, Nasal; Ot, Otooccipital; P, Parietal; Pa, Palatine; Pfr, Postfrontal; Po, Postorbital; Prf, Prefrontal; Pt, Pterygoid; Px, Premaxilla; Q, Quadrate; Sq, Squamosal. The anterior portion of the snout of MNRJ 12124 is broken. Scale bar 1 cm.

**FIGURE S8.** Tarsus of right hindlimb of the UF:Herp:181922 specimen: (a) ventral view; (b) dorsal view. Ta, Tarsal; Mt, Metatarsal. The triangle indicates the unfused epiphysis of the zeugopod. Scale bar 1 cm.

**TABLE S1.** Ct scan parameters.

## Data Availability

Data will be available from the authors upon reasonable request.
