# Supplementary material for: The skeleton of the green Iguana iguana (Squamata: Iguanidae) and its intraspecific morphological variation
Source: Anat Rec (Hoboken). 2025 Dec 17;309(11):3345–74. doi: 10.1002/ar.70108 (PMC13645038; doi:10.1002/ar.70108)
Supplement: Supplementary file 1 [file AR-309-3345-s003.docx]

| **Collection id** | **Voxel size**  **(mm)** | **Current (mA)** | **Energy (kV)** | **Number of slices** |
| --- | --- | --- | --- | --- |
| Ig01 | x, y and z = 0.625 | 400 | 80 | 1,193 |
| Ig02 | x, y and z = 0.625 | 400 | 80 | 257 |
| Ig03 | x, y and z = 0.625 | 400 | 80 | _ |
| Ig04 | x, y and z = 0.625 | 400 | 80 | 1,116 |
| Ig05 | x, y and z = 0.625 | variable | 80 | 474 |
| UF:Herp:181922 | x, y and z =0.137436 | 0.2 | 120 | 2,700 |
